# Intelligent berberine-loaded erythrocytes attenuated inflammatory cytokine productions in macrophages

**DOI:** 10.1038/s41598-024-60103-9

**Published:** 2024-04-23

**Authors:** Zahra Sadat Aghili, Mauro Magnani, Mehdi Ghatrehsamani, Azar Nourian Dehkordi, Seyed Abbas Mirzaei, Mehdi Banitalebi Dehkordi

**Affiliations:** 1 Department of Molecular Medicine, School of Advanced Technologies, Shahrekord University of Medical Sciences, Shahrekord, Iran; 2 Department of Biomolecular Sciences, University of Urbino Carlo Bo, Via Saffi 2, 61029 Urbino, PU Italy; 3 Cellular and Molecular Research Center, Basic Health Sciences Institute, Shahrekord University of Medical Sciences, Shahrekord, Iran; 4 Department of Medical Biotechnology, School of Advanced Technologies, Shahrekord University of Medical Sciences, Shahrekord, Iran

**Keywords:** Berberine, Drug delivery, Erythrocytes, Osmotic fragility, Taguchi methodology, Biological techniques, Biotechnology, Cell biology, Immunology

## Abstract

Erythrocytes are impressive tools for drug delivery, especially to macrophages. Therefore, berberine was loaded into erythrocytes using both hypotonic pre-swelling and endocytosis methods to target macrophages. Physicochemical and kinetic parameters of the resulting carrier cells, such as drug loading/release kinetics, osmotic fragility, and hematological indices, were determined. Drug loading was optimized for the study using Taguchi experimental design and lab experiments. Loaded erythrocytes were targeted to macrophages using ZnCl_2_ and bis-sulfosuccinimidyl-suberate, and targeting was evaluated using flow cytometry and Wright–Giemsa staining. Differentiated macrophages were stimulated with lipopolysaccharide, and the inflammatory profiles of macrophages were evaluated using ELISA, western blotting, and real-time PCR. Findings indicated that the endocytosis method is preferred due to its low impact on the erythrocyte’s structural integrity. Maximum loading achieved (1386.68 ± 22.43 μg/ml) at 1500 μg/ml berberine treatment at 37 °C for 2 h. Berberine successfully inhibited NF-κB translation in macrophages, and inflammatory response markers such as IL-1β, IL-8, IL-23, and TNF-α were decreased by approximately ninefold, sixfold, twofold, eightfold, and twofold, respectively, compared to the LPS-treated macrophages. It was concluded that berberine-loaded erythrocytes can effectively target macrophages and modulate the inflammatory response.

## Introduction

Inflammation is a natural type of “life insurance”, a body-defending system against pathogen invasion. On the other hand, chronic inflammation may contribute to a variety of disorders, such as diabetes, obesity, rheumatoid arthritis, atherosclerosis, and cancer^1–3^. It is also accepted that macrophages play a pivotal role in chronic inflammation in most human disorders^4,5^. Accordingly, there is a strong demand to develop new delivery procedures to target macrophages. Liposomes, dendrimers, nanoparticles, and polymer conjugates are just some of the artificial delivery systems. Unfortunately, synthetic carriers could be very expensive, and their metabolism may result in toxic derivatives^6–10^. Consequently, developing natural drug delivery systems is crucial and a rapidly emerging field^11–13^. Erythrocytes (RBCs; red blood cells) are among the most valuable vascular carriers for promoting the biodistribution, pharmacokinetics, and pharmacodynamics of pharmacotherapeutics. In addition, erythrocytes are considered due to their clinical safety in transfusion and drug loading efficiency^14–18^. Reticuloendothelial system cells (RES) phagocytose aged erythrocytes; therefore, carrier erythrocytes could potentially deliver their payload to RES cells in a targeted manner. Senescent antigen appears on the surface of aged or damaged erythrocytes and induces the binding of autologous IgG, which promotes macrophage clearance of senescent cells^17^. Recent research has shown that berberine hydrochloride, as a pleiotropic chemical, may efficaciously facilitate its anti-inflammatory activities in the immune system by interacting with various signaling molecules and transcription factors such as nuclear factor-κB (NF-κB)^19–21^.

A study indicated that clodronate-loaded engineered erythrocytes selectively targeted to phagocytic cells are able to deplete macrophages. The transient suppression of macrophage functions through clodronate-loaded erythrocytes can be used in many biomedical phenomena and research applications^22^. Also, loaded erythrocytes have been shown to be potentially beneficial for the delivery of aminoglycoside antibiotics in macrophages^23^. In another study, the anti-tumor efficacy of erythrocytes encapsulating zoledronate was assessed in a mouse model of mammary cancer. The findings indicate the suitability of zoledronate-loaded erythrocytes as pharmacological agents as part of a therapeutic strategy in cancer treatment, targeting tumor-associated macrophages^24^. A novel antisense 14-mer anti-inducible nitric oxide synthase (iNOS) was loaded in erythrocytes. Loaded erythrocytes were targeted to macrophages using an in vitro opsonization generated by ZnCl_2_ and bis-sulfosuccinimidyl-suberate (BS^3^). The efficiency of this delivery strategy is demonstrated by decreased NO generation and iNOS protein expression within macrophages^25^. To improve the hypolipidemia efficacy and reduce side effects, the researchers encapsulated berberine into erythrocytes to explore its sustained-release effect by the hypotonic pre-swelling method. The results indicated that this strategy is a promising delivery system that can achieve long circulation and sustained release of berberine with hypolipidemic effect^26^.

The aim of this study is to investigate the use of erythrocytes as a natural drug delivery system for targeted delivery of berberine to macrophages. By encapsulating berberine within erythrocytes, we aim to overcome the limitations associated with its poor bioavailability and enhance its therapeutic efficacy in combating inflammation. The hypothesis of this study is that berberine-loaded erythrocytes will exhibit improved stability, enhanced accumulation in macrophages through RES targeting, and increased anti-inflammatory effects on LPS-induced inflammatory responses in PMA-differentiated U937.

## Results

### Berberine loading procedure, quantification, and characterization

**Figure 1:**
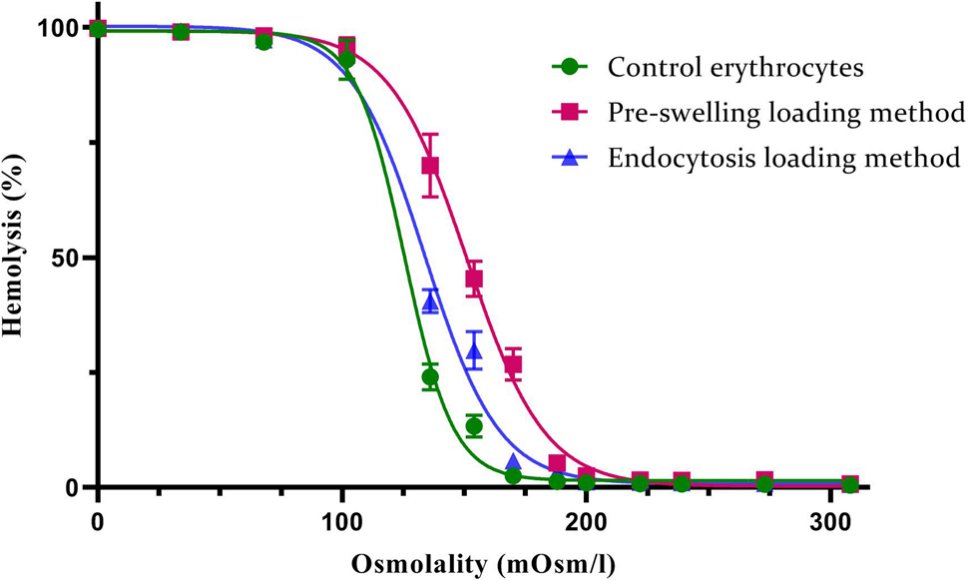
Erythrocytes’ osmotic fragility plots. Hemolysis quantification of control erythrocytes and berberine-loaded erythrocytes in pre-swelling and endocytosis methods was measured, and corresponding equations (Y = 2E−05X^3^ − 0.01X^2^ + 0.12X + 104.46; Y = 2E−05X^3^ − 0.01X^2^ + 0.61X + 96.39; Y = 2E−05X^3^ − 0.01X^2^ + 0.31X + 101.54) were fitted on the charts, and a square regression coefficient of 0.91, 0.96, and 0.94 were obtained, respectively. MCF_50_ values of 134.20 ± 2.51, 138.34 ± 2.66, and 143.62 ± 6.72, mOsm/l produced 50% hemolysis of control erythrocytes, berberine-loaded erythrocytes in endocytosis method, and pre-swelling method, respectively. The statistical analysis of the data in MCF_50_ values indicates significant differences between the control erythrocytes, and loaded erythrocytes (p ≤ 0.001).

**Figure 2:**
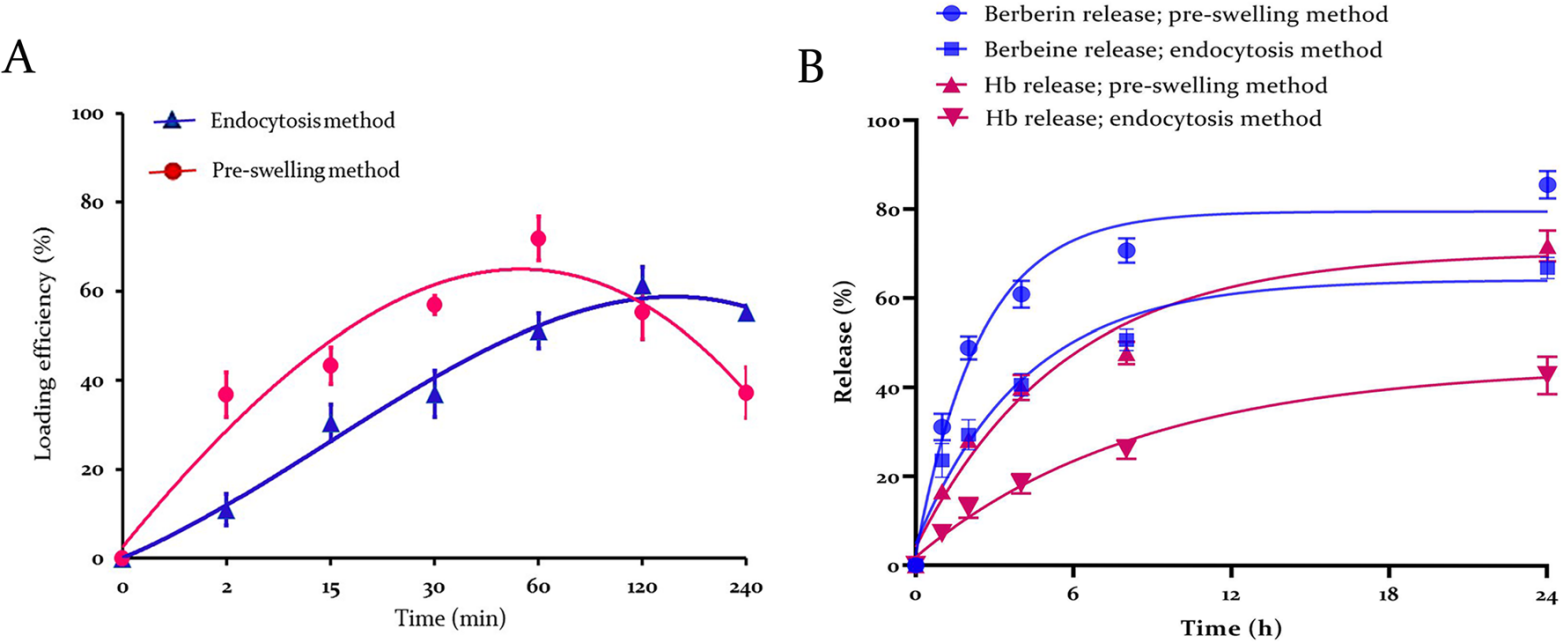
Berberine loading and release profiles and erythrocyte hemolysis pattern. Maximum drug loading was attained after 60 min for the pre-swelling method and 120 min for the endocytosis method (**A**). In-vitro berberine and hemoglobin releases from loaded erythrocytes at pH 7.4. (**B**) Revealed that the endocytosis method might result in minor injury to the cell membrane.

Initially, berberine quantification was calibrated on HPLC, and a linear equation (Y = 25.77X + 922.13) was fitted between the berberine concentrations and the chromatogram areas, and a square regression coefficient of 0.99 was obtained, as shown in Supplementary Fig. S1. MCF_50_ values of 134.20 ± 2.51, 138.34 ± 2.66, and 143.62 ± 6.72 mOsm/l displayed 50% hemolysis of the controls, berberine-loaded erythrocytes with the endocytosis method, and pre-swelling method, respectively, as shown in Fig. 1. Maximum berberine loading for the pre-swelling method was 71.85 ± 4.95% after 60 min. The endocytosis method was saturated after 120 min with 61.43 ± 4.09% of berberine. Berberine loading kinetics for pre-swelling and endocytosis methods follow Y =  − 0.27T^3^ − 1.38T^2^ + 32.13T − 27.83 and Y =  − 0.44T^3^ + 3.91T^2^ + 3.24T − 6.63 equations with regression coefficients of 0.99 and 0.94, respectively. Where T is the time in minutes (Fig. 2A).

**Figure 3:**
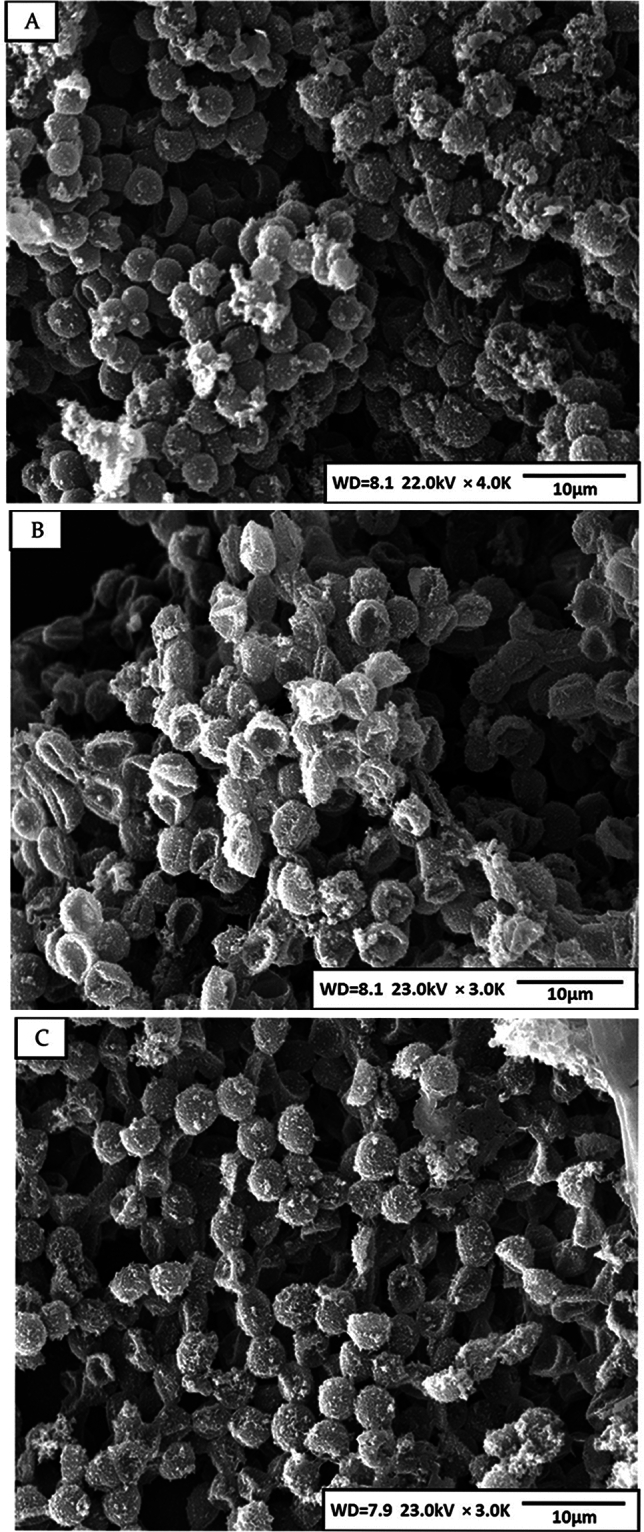
SEM pictures for control erythrocytes (**A**), berberine loaded erythrocytes in pre-swelling method (**B**), and endocytosis methods (**C**). Images represented no visible changes in erythrocytes’ size and morphology after the maximum loading conditions (1500 μg/ml berberine concentration, at 25 °C for 120 min, and pH 7.4).

Loading of berberine through the pre-swelling method showed an increase in the osmotic fragility of the loaded erythrocytes compared to the endocytosis method. Furthermore, an initial burst release of berberine was attained over the first few hours in both methods. The pre-swelling method rapidly leaked off about 50% of the loaded berberine, while the amount of berberine released in the endocytosis method was slightly lower. Release profiles were almost saturated and plateaued during the first 8 h. Maximum berberine release from erythrocytes was achieved after 24 h and near 85% and 66% in the pre-swelling and endocytosis methods, respectively. In addition, a remarkable correlation between the release of berberine and the hemolysis profile of erythrocytes can be observed (Fig. 2B). The endocytosis method efficiently loaded berberine, and it also protected erythrocytes from hemolysis. Erythrocyte volume was not significantly affected by drug loading with any loading methods, and MCV values were all within the normal range. However, MCH and MCHC levels decreased meaningfully when the erythrocytes were subjected to the drug loading procedures. These changes were milder in erythrocytes prepared by the endocytosis method (Table 1). SEM indicated that the maximum loading procedures did not have any significant impacts on the morphology of erythrocytes (Fig. 3).

**Table 1:** Physicochemical and kinetic parameters for berberine loading in human erythrocytes using pre-swelling and endocytosis methods.

	Control erythrocytes	Berberine loading; pre-swelling method	Berberine loading; endocytosis method
Loading kinetics; R-square (T is the time in minute)	–	Y = − 0.27T^3^ − 1.38T^2^ + 32.13T − 27.83; R^2^ = 0.99	Y = − 0.44T^3^ + 3.91T^2^ + 3.24T − 6.63 R^2^ = 0.94
Maximum berberine loading (%)	–	71.85 ± 4.95%; after 60 min	61.43 ± 4.09%; after 120 min***
Maximum berberine loading (μg/ml)	–	179.62 ± 5.80; after 60 min	153.57 ± 5.02; after 120 min*
Median corpuscular fragility; R-square (X is salt concentration mOsm/l)	Y = 2E−05X^3^ − 0.01X^2^ + 0.12X + 104.46; R^2^ = 0.91	Y = 2E−05X^3^ − 0.01X^2^ + 0.61X + 96.39; R^2^ = 0.96	Y = 2E−05X^3^ − 0.01X^2^ + 0.31X + 101.54; R^2^ = 0.94
MCF_50_ (mOsm/l)	134.20 ± 2.51	143.62 ± 6.72***	138.34 ± 2.66**
MCV (fl) after loading	87. 60 ± 0.43	87.60 ± 1.60	86.56 ± 1.48
MCH (pg) after loading	29.7 ± 0.85	26.10 ± 1.20***	27.53 ± 1.53**
MCHC (g/dl) after loading	32.66 ± 0.40	28.61 ± 2.69***	29.53 ± 1.75**
Cell recovery (%)	–	80.09 ± 8.63	93.50 ± 6.99*
Hb release (%) after loading	–	71.86 ± 5.89	42.86 ± 3.98*
Hb release kinetics (%); R-square (T is time in hour)	–	Y = 0.87T^3^ − 8.73T^2^ + 38.05T − 30.59; R^2^ = 0.99	Y = 0.56T^3^ − 4.86T^2^ + 18.62T − 14.51; R^2^ = 0.99
Berberine release kinetics; R-square (T is time in hour)	–	Y = 0.95T^3^ − 11.99T^2^ + 60.22T − 49.13; R^2^ = 1.00	Y = 0.92T^3^ − 10.06T^2^ + 43.99T − 34.11; R^2^ = 0.99
Maximum berberine release (%) after 24 h	–	85.61 ± 9.06	66.92 ± 8.20**

The statistical analyses were performed with a one-way analysis of variance, and p ≤ 0.001, p ≤ 0.01, and p ≤ 0.05 are represented as (***), (**), and (*).

### Optimization of the endocytosis method for berberine loading

**Figure 4:**
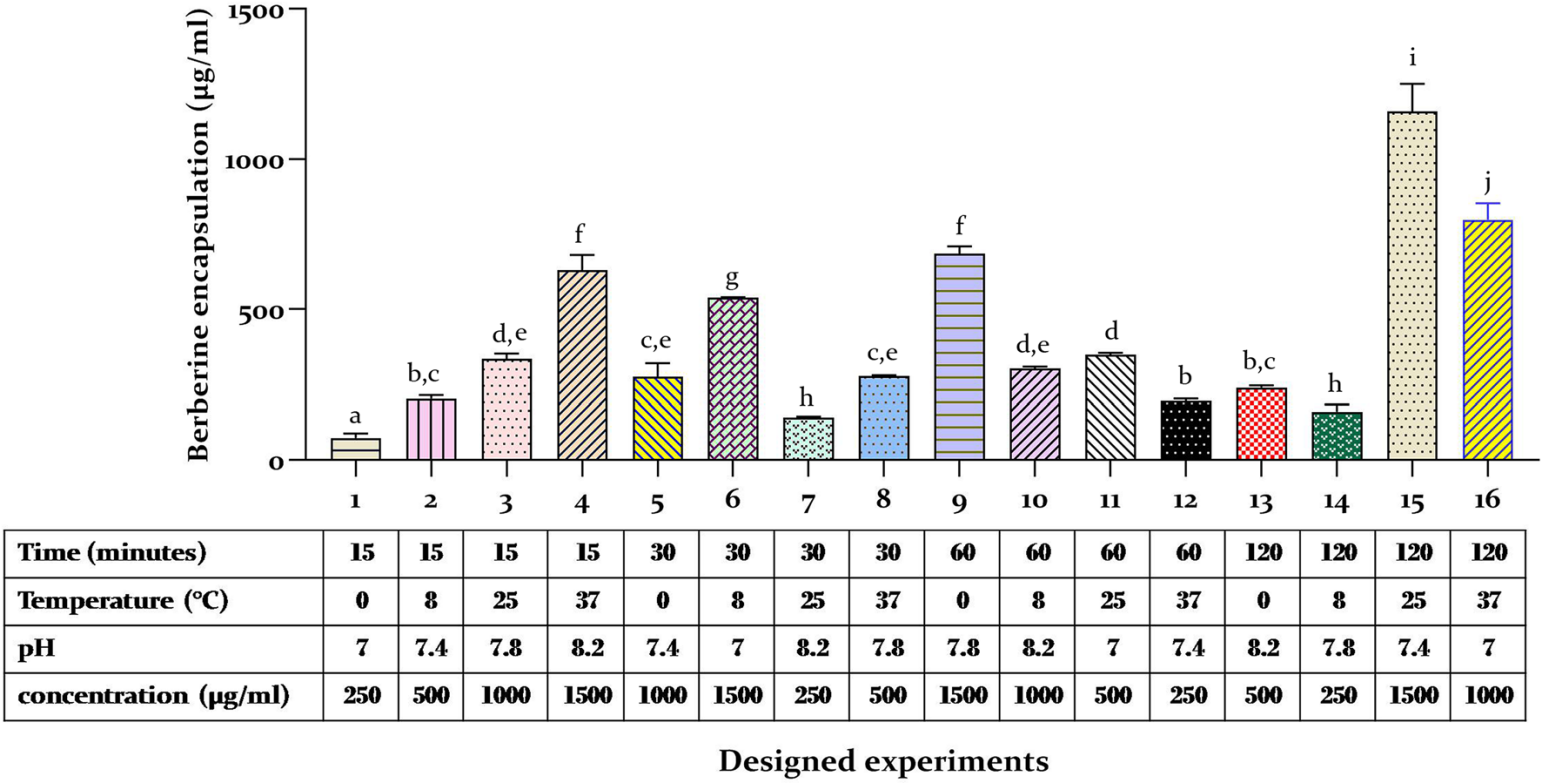
The graph shows the concentration of berberine loading in erythrocytes by the endocytosis method in 16 designed experiments using the Taguchi method. The maximum berberine concentration was achieved through the incubation of erythrocytes with a concentration of 1500 µg/ml berberine at a temperature of 25 °C and a pH of 7.4 for 2 h. Treatments with the same letter are not significantly different at a certain level of significance (one-way ANOVA, homogeneity of variance, p > 0.05).

Figure 4 displays the findings for all response variables for the designed trial in Table 2 and Supplementary Table S1. It is clear that there were significant differences in the responses across all trials. The concentration of berberine loading in erythrocytes varied from 70.36 to 1160.09 µg/ml (~ 16-fold difference). Meanwhile, the maximum berberine-loaded amount was obtained under the 15th trial. The modeling results indicated that the maximum concentration of berberine loading appeared when the experimental conditions were: temperature (25 °C), time (120 min), medium pH (7.4), and berberine concentration (1500 µg/ml). Supplementary Figure S2 depicts the main effects (the average of the obtained results at each given level) to provide insight into each factor’s individual effects at the analyzed levels. Berberine loading improved with increases in berberine concentration, incubation temperature, and time. While berberine concentration represented the major influence, pH levels had the least effect on the loading responses. Taguchi DOE calculated the severity index (SI, the interaction percentage between two elements). Supplementary Figure S3 reveals the main effect interaction plot and severity index of factors on berberine loading. The most significant interaction, SI = 41.41%, was estimated between temperature and pH. According to the ANOVA in Supplementary Table S2, berberine concentration was the most significant factor in optimizing berberine loading in erythrocytes (equal to 75.83%). Time and temperature contributions are 9.76% and 9.00%, respectively. pH has a null effect on berberine loading in erythrocytes. Taguchi DOE presented optimum culture conditions for each factor and contributed to attaining the highest drug loading. Supplementary Table S3 shows the optimal conditions, these conditions were a berberine concentration of 1500 µg/ml, an incubation time of 120 min, and an incubation temperature of 37 °C. The anticipated quantity of berberine loading in optimum conditions was estimated at 1367.73 µg/ml by the Taguchi method. Performing a real set of loading experiments under the optimized condition led to 1386.68 ± 22.43 µg/ml, confirming and validating the computational model. It is evident from the results that the optimized conditions enhanced berberine loading. Supplementary Figure S4 displays the plot of process capability (variation reduction plot), which indicates the difference between the current and improved conditions in terms of deviation from the mean. The improved condition exhibits a smaller standard deviation in comparison to the current condition. As in this study, the standard deviation in the current condition decreased from 384.88 to 59.96 in the improved condition.

**Table 2:** Factors for optimization of berberine loading in erythrocytes and their assigned levels.

Factors	Dimensions	Level 1	Level 2	Level 3	Level 4
Time	Min	15	30	60	120
Temperature	°C	0	8	25	37
pH	–	7	7.4	7.8	8.2
Berberine concentration	μg/ml	250	500	1000	1500

### Quorum-sensing signals between macrophages and berberine-loaded erythrocytes

**Figure 5:**
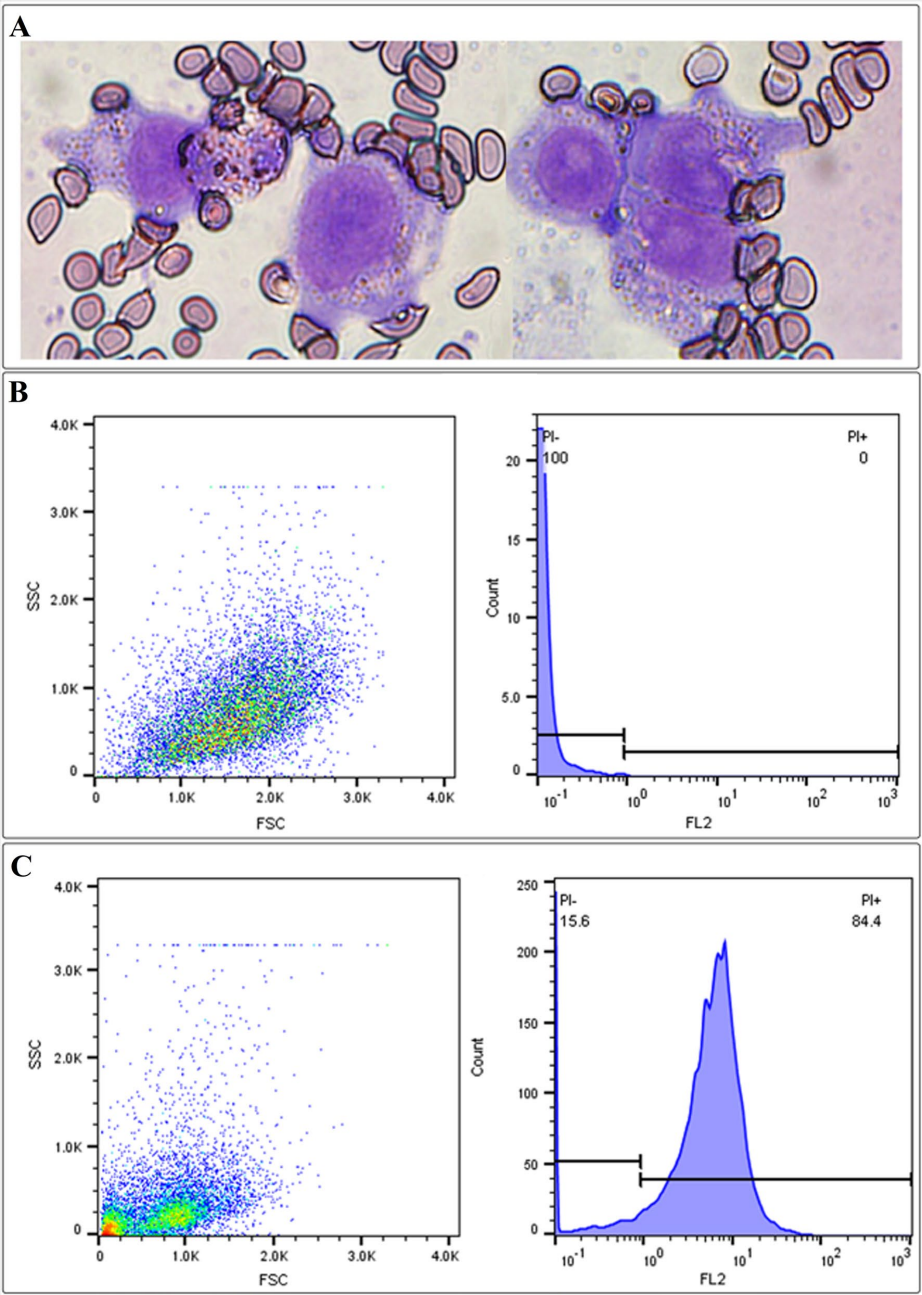
Wright–Giemsa staining and flow cytometer analyses for activated macrophages. (**A**) Represented phagocytosis activity of macrophages with formation of primary podosomes and filopodia-like protrusions that surround erythrocytes at bright field microscope at × 100 magnifications. (**B**) Was the control FSC/SSC dot blot and the FL2 histogram using FL2 filter on the flow cytometer. (**C**) Represented appropriate counts levels of macrophages that actively phagocytosed the labeled-erythrocytes.

**Figure 6:**
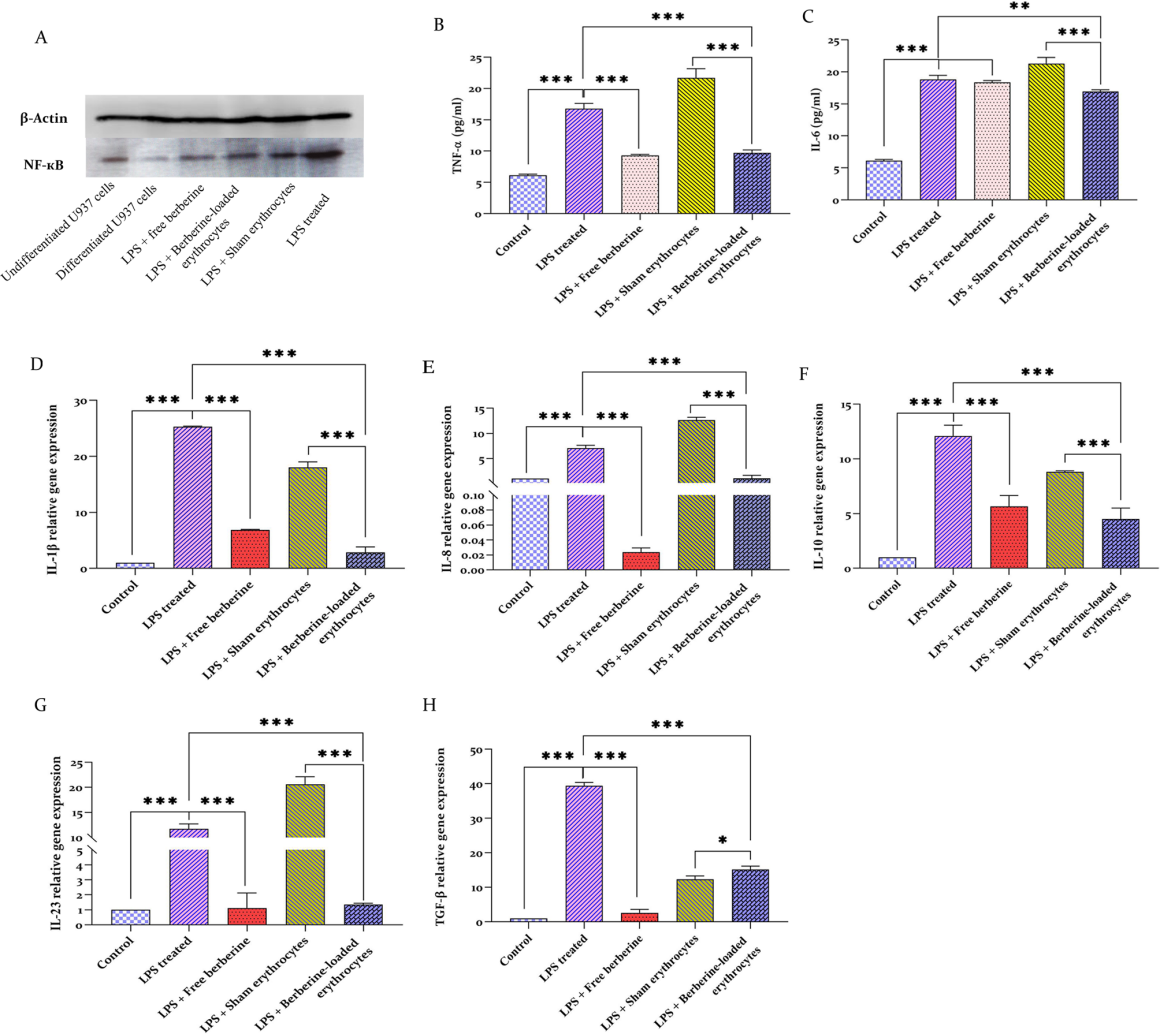
Quantitative analyses of inflammatory cytokine transcripts and proteins. (**A**) Western blotting represented that p-NF-κB P65 protein was increased in LPS-treated macrophages and decreased in berberine pretreatment. ELISA verified a decreased level of TNF-α (**B**) and IL-6 (**C**) in berberine-treated macrophages. Transcript level of IL-1β (**D**), IL-8 (**E**), IL-10 (**F**), IL-23 (**G**) and TGF-β (**H**) represented a significant reduction in berberine-treated macrophages. One-way ANOVA was used to determine the significance between the groups. Western blot control is non-treated results of differentiated U937. Sham erythrocytes mean berberine-free erythrocytes. p ≤ 0.001 (***), p ≤ 0.01 (**) and p ≤ 0.05 (*) were stated significant.

Primarily, erythrocyte targeting to macrophages and erythrophagocytosis were confirmed by Giemsa staining and flow cytometry. Macrophages in Fig. 5A started the phagocytosis process by podosome formation and filopodia-like protrusion during incubation with loaded erythrocytes. Red fluorescently labeled erythrocytes with DiI were phagocytosed by macrophages and subsequently counted by flow cytometer. Figure 5B,C histograms represented high counts of stained macrophage population (events) on the FL2 channel at 488 nm excitation and 585 nm emission for the sample in comparison to the control. Western blot analyses revealed downregulation and a meaningful reduction in the band intensity of NF-κB P65 in berberine-treated groups, free berberine, and berberine-loaded erythrocytes (Fig. 6A, Supplementary Fig. S5). Although LPS increased TNF-α (16.76 ± 0.85 pg/ml) and IL-6 (18.82 ± 0.68 pg/ml) secretions in macrophages by several folds, berberine and berberine-loaded erythrocytes mostly diminished inflammation responses (Fig. 6B,C). Real-time PCR using appropriate primers listed in Supplementary Table S4 showed that IL-1β (25.28-fold), IL-8 (7.06-fold), IL-10 (12.08-fold), IL-23 (11.71-fold), and TGF-β (39.39-fold) were upregulated in LPS-treated macrophages. Pretreatment with berberine-loaded erythrocytes significantly contracted IL-8 (6.68-fold), IL-1β (8.90-fold), IL-10 (2.68-fold), IL-23 (8.67-fold), and TGF-β (2.60-fold) transcription (Fig. 6D–H). Pretreatment with free berberine also inhibited inflammatory cytokine transcriptions.

## Discussion

Berberine loading kinetics through pre-swelling method revealed that the maximum amount of drug loading (71%) is attained after 60 min. This loading was higher than most of formerly published results, such as amphotericin B (16%)^27^, ambroxol hydrochloride (61%)^28^, pravastatin chitosan nanogels (36%)^29^, and methotrexate (60%)^30^. On the other hand, 61% berberine loading in endocytosis method was almost comparable with previously published data for pravastatin (94%)^31^, artemether (14%)^32^, metformin (90%)^33^ and salbutamol (68%)^34^. As revealed in Fig. 2A, once the maximum amount of berberine was loaded, a portion of the drug gradually departed from cells as a results of diffusion and hemolysis. The pre-swelling method resulted in a higher amount of berberine loading and also higher hemolysis; therefore, the loading graph decreased more rapidly, after reaching the top. On the other hand, pre-swelling method increased the osmotic fragility of loaded erythrocytes compared to endocytosis method (Fig. 1). It has been demonstrated that paclitaxel entrapment using pre-swelling method greatly enhances the osmotic fragility of the cells, and that the population of paclitaxel-loaded cells exhibits greater heterogeneity in terms of cell membrane resistance to variations in external osmotic pressure than do normal unloaded erythrocytes^35^ .Similarly, an additional investigation found that the osmotic fragility curves shifted from an S-shape for control erythrocytes to a virtually linear shape for erythrocytes that had been loaded with drugs^36^.

Hematological parameters, which are routinely assessed in clinical hematology tests, can provide valuable insights into the physiological condition of erythrocytes. The findings of our study don’t demonstrated significant changes in erythrocyte volume, as indicated by the MCV values, resulting from the entrapment process during sham and berberine loading. Furthermore, both the MCH and MCH decreased following the exposure of erythrocytes to the loading procedure, regardless of whether it was sham or berberine loaded erythrocytes. The reduction in hemoglobin content in erythrocytes during the loading procedure was expected due to the inherently destructive nature of the procedure; consistent with previous investigations^35,36^. However, it is noteworthy that the changes in MCH and MCHC levels were milder in erythrocytes prepared using the endocytosis method. This finding implies that the endocytosis method may offer certain advantages in terms of preserving the erythrocyte’s hemoglobin content and concentration during the drug loading process. Preserving the hemoglobin content and concentration is crucial for maintaining the erythrocytes’ physiological function. Therefore, the milder changes observed with the endocytosis method suggest that this approach may be better suited for minimizing potential alterations in erythrocyte functionality compared to other method.

Release kinetics in Fig. 2 represent a burst berberine outflow in the first few hours that might be a rapid release of berberine bound to the membrane surface^31^. Endocytosis was a gentle method that increased drug retention and erythrocyte survival, so berberine was released more slowly in this method due to a minor injury to the erythrocyte membranes. In contrast, the possible explanation for the pre-swelling method’s lack of control over drug release implicates membrane integrity due to hypotonic pressure^36^. In addition, there was a remarkable correlation between the berberine release profile and hemoglobin release that is consistent with those of other drugs, such as pravastatin^31^, primaquine^37^, terpene-indole alkaloids^38^, and paclitaxel^35^. Erythrocyte membrane must balance tensile strength and deformability to pass through narrow capillaries^39^. The erythrocyte membrane integrity must be maintained to prevent intravascular hemolysis and the release of free hemoglobin, which can damage the kidneys and endothelium^11^. According to our results, it was reasonable to conclude that drug loading into erythrocytes by the endocytosis method is preferred because it has little impact on the erythrocyte's structural integrity and is therefore more suitable for subsequent manipulations.

Research has corroborated the necessity of specific parameters such as temperature, time, pH, and drug concentration to enhance the efficacy of drug loading within erythrocytes^35,40–42^. Qualitek software provides an ANOVA table that describes the actual contributions of the factors. Berberine concentration was the most significant factor in optimizing berberine loading in erythrocytes (equal to 75.83%) and pH was presented as the lowest contributor. F-ratio values above 3.00 were more than 90% significant (Supplementary Table S2). Taguchi DOE predicted optimal culture conditions that were a berberine concentration of 1500 µg/ml, an incubation time of 120 min, and an incubation temperature of 37 °C. The expected amount of berberine loading in such a condition was 62.72 units based on signal-to-noise (S/N) ratios. So, optimization enhances berberine loading by around 140% rather than the grand average (Supplementary Table S3, Supplementary Fig. S3). Real experiments resulted in 1386.68 µg/ml loading and confirmed the predicted conditions. This result was found to be comparable to the predicted loading of 1367.73 µg/ml, thereby providing evidence for the validity of the methods^43–45^.

Following macrophage exposure to the LPS challenge, there was a significant upregulation in the production of pro-inflammatory cytokines in macrophages. TNF-α, IL-1β, IL-23, IL-8, and IL-6 are widely recognized as key indicators of the inflammatory response. These molecules are implicated in the initiation of the NF-κB signaling pathway, which subsequently stimulates the transcription of various factors that exacerbate the inflammatory response^46,47^. Therefore, it is crucial to inhibit the expression of these cytokines. Figure 6 indicated that berberine, either in its free form or loaded in erythrocytes, led to a noteworthy reduction of inflammatory cytokines at the mRNA and protein levels in macrophages. Berberine-loaded erythrocytes were strong enough to block the inflammation responses in the molecular evaluations. The research findings indicate that the addition of LPS resulted in an increase in two anti-inflammatory cytokines, namely TGF-β and IL-10. It has been shown that these anti-inflammatory cytokines may operate as a negative regulator of inflammation. As a result, an inflammatory stimulus also induces its negative regulator, allowing the process to be controlled and excessive damage to host tissues to be avoided^48,49^.

This erythrocyte-based drug delivery system has a number of benefits over current methods for targeted delivery of berberine to macrophages. Erythrocytes have been extensively studied for their biocompatibility and safety in clinical use. The use of erythrocytes as carriers for berberine offers enhanced biocompatibility and reduced toxicity compared to synthetic delivery systems. Synthetic carriers often carry the risk of toxicity and adverse side effects. In contrast, erythrocytes have a long-standing safety record and are commonly used for transfusion purposes. Targeting macrophages is crucial in chronic inflammation, as these immune cells play a central role in disease progression. The erythrocyte-based drug delivery system takes advantage of the natural phagocytic activity of macrophages towards aged or damaged erythrocytes. This results in the preferential uptake of berberine-loaded erythrocytes by macrophages. This targeted approach increases the local concentration of berberine at the site of inflammation, potentially enhancing its anti-inflammatory efficacy. Another significant advantage of this system is the improved stability and prolonged circulation of berberine. Berberine is known for its poor bioavailability and short half-life, limiting its therapeutic potential. Encapsulating berberine within erythrocytes causes prolonged circulation and enhances the availability of berberine for uptake by macrophages, improving its therapeutic efficacy. Furthermore, the high drug loading efficiency of erythrocytes is another valuable attribute of our system. Erythrocytes have a large surface area and can accommodate a substantial amount of cargo. This high drug loading efficiency allows for the loading of a significant quantity of berberine within the erythrocytes, maximizing the payload delivered to the target cells. While our study demonstrates the promising advantages of the erythrocyte-based drug delivery system, it is important to acknowledge certain limitations and assumptions. For instance, further investigations are required to optimize the loading efficiency and release kinetics of berberine from erythrocytes. Additionally, the long-term stability and potential immunogenicity of berberine-loaded erythrocytes need to be thoroughly evaluated.

In conclusion, an erythrocyte-based drug delivery system for berberine has been developed to target macrophages and increase drug biocompatibility. After optimization of the study, results indicated that human erythrocytes effectively loaded berberine. These findings indicated that berberine-loaded erythrocyte is a qualified carrier to attenuate the secretion of inflammatory factors by targeting macrophages, and it will have a suitable candidate in the future.

## Materials and methods

### Reagents

Adenosine 5-triphosphate (ATP), phorbol myristate acetate (PMA), LPS, bovine serum albumin (BSA), and bis-(sulfosuccinimidyl)-substrate (BS_3_) were from Sigma-Aldrich (St. Louis, MO). RPMI 1640 medium and FBS were purchased from Gibco (Carlsbad, CA). Ficoll was obtained from Lymphodex (Inno-Train, Germany). Berberine hydrochloride, anti-NF-κB p65, anti-β-Actin, and m-IgGκ BP-HRP antibodies were obtained from Santa Cruz Biotechnology (Santa Cruz, CA, USA). All other solvents and salts were purchased from Sigma-Aldrich and were of biological grade.

### Ethics statement

This study was approved and conducted according to the institutional ethical guidelines of the ethical committee of Shahrekord University of Medical Sciences (IR.SKUMS.REC.1399.089). All procedures were carried out in accordance with the Declaration of Helsinki. Written informed consent was given by the donors.

### Berberine quantification and loading efficiency/capacity

HPLC was used for berberine quantification in erythrocytes. Briefly, 900 µl of double distilled water was added to 100 µl of the final berberine-loaded suspension. The suspension was boiled for five min, filtered through 0.22 µm cellulose acetate filters, and then injected into the HPLC (Agilent Technologies 1100 series, Waldbronn, Germany) equipped with a C18 column (25 cm × 4.6 mm, Agilent) and a UV detector (VWD, Agilent Technologies). The flow rate was set at 1 ml/min, and the berberine picks were recorded at 210 nm. The mobile phase consisted of buffer-A (10 mM KH_2_PO_4_, pH 5.0) and buffer-B (buffer-A containing 30% v/v acetonitrile). Elution started with 100% buffer-A for 5 min. Then, gradually replaced with 100% buffer-B over 10 min, and hold for another 8 min. A serially diluted pure berberine from 0 to 100 µg/ml was used as a quantification standard curve. Loading efficiency (LE %) represents the minimal wastage of precious berberine and, could be measured as (LE %) = (W_t_ ÷ W_i_) × 100. Where W_t_ is the total berberine amount inside erythrocytes and W_i_ is the total added berberine^50^.

### Physicochemical characteristics of carrier erythrocytes

Variables such as fragility behavior, hematological Indices and, morphologies of the erythrocytes demonstrate how the loading procedure affects structural changes in berberine-loaded erythrocytes in pre-swelling and endocytosis methods. To determine the osmotic fragility of erythrocytes, 100 µl of packed cells were placed in a series of tubes containing 900 µl sodium chloride solutions ranging from 0 to 308 mOsm/l. After gentle mixing, the erythrocyte suspensions were incubated for 30 min at 37 °C and then centrifuged for 10 min at 300×*g*. The amount of hemoglobin released was measured using colorimetric analysis at 540 nm using the following equation: % hemolysis = 100 × (sample absorbance ÷ positive control absorbance), where hemolysis in water is the positive hemolysis. Median corpuscular fragility-50 (MCF_50_) is the salt solution that induces 50% hemolysis of erythrocytes^51^. Besides, the number of different parameters like the mean corpuscular volume (MCV), the mean corpuscular hemoglobin (MCH), and the mean corpuscular hemoglobin content (MCHC) were determined using a Coulter counter-based instrument (Sysmex XT 1800i Hematology Analyzer; Japan)^52^. Furthermore, control and berberine-loaded erythrocytes were added to a tube containing 1 ml of 2.5% glutaraldehyde and fixed for 30 min. Then they were gently mixed in 1 ml of post-fixation solution (0.4% w/v potassium permanganate and 0.6% w/v potassium dichromate) for 5 min. Next, samples were dehydrated at gradient concentrations of ethanol from 30 to 100% for 5 min, and the solvent was replaced with isoamyl acetate for 30 min^53^. After being coated with gold particles using a Sputter Coater at 18 mA for 1 min, erythrocytes were pictured by a scanning electron microscope (SEM).

### Berberine loading study in erythrocytes

Hypotonic pre-swelling and endocytosis methods were both examined for berberine loading in fresh erythrocytes. Blood samples from healthy donors were collected in heparinized tubes and centrifuged at 300×*g* at 4 °C for 10 min. The red cell fraction was collected and washed three times with a neutral isotonic sodium chloride solution (0.9% w/v). Then, erythrocytes were resuspended in an isotonic sodium chloride solution to obtain 70% hematocrit. In the pre-swelling procedure, the erythrocytes’ osmotic fragility was initially assessed to achieve the optimal hypo-osmotic solution tonicity for drug loading. Then, 200 µl erythrocyte suspension was added to 800 µl of berberine-containing hypo-osmotic solution (102 mM NaCl, 250 μg/ml berberine at pH 7.4). Next, the suspension was incubated at ambient temperature for 15 min. Afterward, erythrocytes were resealed by rapidly adding 100 µl of hypertonic NaCl (6% w/v) and gentle inverting. Cells were reannealed for 30 min at 37 °C. Finally, loaded erythrocytes were washed twice with an isotonic sodium chloride solution to remove excess drugs and released hemoglobin^38^. For the endocytosis method, 200 µl erythrocyte suspension at a hematocrit of 70% was added to 800 µl isotonic sodium chloride solution containing 2.5 mM ATP, 2.5 mM CaCl_2_, 2.5 mM MgCl_2_, and 250 μg/ml berberine at pH 7.4. They were gently mixed and incubated at ambient temperature for 15 min; the pores were then resealed by replacing the erythrocyte environment with an isotonic sodium chloride solution for 30 min at 37 °C. Finally, the erythrocytes were harvested and washed twice with an isotonic sodium chloride solution^31^. The percentage of cell recovery in both methods was calculated by counting the number of erythrocytes in a hemocytometer before and after berberine loading^54^.

### In-vitro release kinetics for berberine and hemoglobin

Berberine and hemoglobin release from loaded erythrocytes were evaluated as follows: 0.5 ml of packed, loaded erythrocytes was diluted to 4.5 ml in PBS buffer with a pH of 7.4. The suspension had been thoroughly mixed by several mild inversions. Then, the suspension was divided into five portions in Eppendorf tubes and incubated at 37 °C under gentle seesaw shaking. One of the samples was taken and centrifuged at 300×*g* for 5 min at the start of the test and intervals of 1, 2, 4, 8, and 24 h. The supernatants were divided into 100 µl for the drug assay. Additionally, a spectrophotometer measured the absorbance of 200 µl of the supernatant at 540 nm. The amount of hemoglobin released was calculated concerning the fully lysed sample^31^.

$$\text{Berberine release} (\text{\%})=[1-\left(\frac{\text{Amount of loaded berberine in RBCs at time point}}{\text{Initial amount of loaded berberine in RBCs }}\right) ] \times 100.$$

### Optimization of endocytosis loading using Taguchi model

The optimization approach used in this work was separated into four phases: experimental design, experimentation, software analysis, and result validation. Each step has a different objective and is interrelated in succession to carry out the overall optimization strategy^38^. In the first phase, crucial factors such as temperature, time, pH, and drug concentration were arranged that may influence drug loading in erythrocytes (Table 2). An L16 orthogonal array (which displays 16 experimental tests) was chosen for the four levels of factor variation (Supplementary Table S1). The second phase starts with the endocytosis experiments. All 16 designed trials were tested in-vitro, as previously described. Data analyses were performed for the third phase, Qualitek-4 software, version 4.82.0 (Nutek Inc., MI, USA), was used for an automatic design and statistical analysis of the results, such as determining the influence of individual factors on drug loading in erythrocytes, their performance at optimum conditions, and an approximation of their performance at optimal results. Finally, the results were validated in the fourth phase. Drug loading in erythrocytes was performed to validate the optimized methodology using optimized culture conditions. To determine the variation between the experiment results and the desired values, the mean squared deviations (MSDs) is calculated with the help of the following equation (greater is better). So, n stands for experiment repeats, and Yi is outcomes^55^.

$${\text{MSD}}=\frac{1}{n}{\sum }_{i=1}^{n}{\left(\frac{1}{{y}_{i}}\right)}^{2}.$$

### Surface modification of erythrocytes, targeting toward macrophages

Macrophage targeting was accomplished by clustering of band 3 membrane proteins on loaded erythrocytes. Band 3 proteins promote IgG opsonization and, finally, phagocytosis via the CR1 complement receptor. Briefly, berberine-loaded erythrocytes and sham erythrocytes (berberine-free erythrocytes) were gently resuspended at 10% hematocrit in 1 ml of an isotonic salt solution containing 1 mM ZnCl_2_ and 1 mM BS^3^ for 15 min at ambient temperature. Cells were then harvested at 300×*g* for 5 min and washed once in an isotonic salt solution containing 10 mM ethanolamine (pH 7.4) and once in an isotonic salt solution containing 1% (w/v) bovine serum albumin. Subsequently, erythrocytes pellet were resuspended with a hematocrit of 30% in autologous plasma for 60 min at 37 °C to promote IgG binding. Finally, erythrocytes were washed once in an isotonic salt solution containing 2% (w/v) BSA and rinsed in a washing isotonic salt solution^33^.

### Differentiation induction and phagocytosis assay

Human monocytic U937 cells (5 × 10^5^ cells/ml) were seeded into a 6-well plate in RPMI-1640 medium supplemented with 10% (v/v) heat-inactivated FBS, 100 U/ml penicillin, 100 μg/ml streptomycin, and 2 mM l-glutamine. 100 nM of PMA for 48 h was supplemented to the media for differentiating the monocytic cells into macrophages if needed. The phagocytosis of erythrocytes by differentiated macrophages was verified by a flow cytometer (CyFlow Space®, Münster, Germany) using the FL2 channel. Briefly, 10 μl of DiI fluorescent dye (1 mg/ml in ethanol) was added to 1 ml of targeted erythrocyte suspension and incubated at 37 °C for 30 min. DiI-linked erythrocytes were harvested, washed with RPMI-1640 three times, and finally harvested. The phagocytosis assay was conducted for 8 h, adding DiI-linked erythrocytes into differentiated macrophages (10 μl targeted DiI-packed erythrocytes per 5 × 10^5^ macrophages) in a phagocytosis medium containing RPMI-1640 medium supplemented with 10% FBS in a humidified 5% CO_2_ incubator at 37 °C. Then, 4 ml of the cell suspension was stratified on Ficoll® and henceforth centrifuged for 20 min at 600×*g* to separate macrophages from non-phagocytosed erythrocytes. Afterward, macrophages were resuspended in the phagocytosis medium after being washed once with the RPMI-1640 medium. The data were analyzed using FlowJo software (version 7.6.1) in comparison to the control samples^56^. Wright-Giemmsa was also used to stain stratified macrophages. A 10 μl aliquot of the cells was spread on a slide to produce a smear, which was dried at 37 °C for 20 min. Slides were fixed for 5 min in absolute methanol at room temperature and air dried. Smears were dyed using Wright–Giemsa solution according to the standard kit procedure and photographed by a light microscope at ×100 magnification^57^.

### Inflammatory genes quantification using real-time PCR

Fresh berberine-loaded erythrocytes were prepared with the maximum loading conditions (1500 μg/ml berberine concentration, at 25 °C for 120 min, and pH 7.4). Differentiated macrophages were treated for 24 h with berberine-loaded erythrocytes (100 erythrocytes per macrophage is the ratio) or 250 µg/ml free berberine (as a positive control) and then stimulated with 1 µg/ml LPS for 18 h. Untreated macrophages and also treated macrophages with empty opsonized erythrocytes (erythrocytes that had undergone the loading method without berberine and subsequently been opsonized) were considered negative controls in the experiment. Next, Ficoll-isolated macrophages were washed with ice-cold PBS, and TRIzol reagent (Invitrogen, CA, USA) was used to isolate total RNA. Real-time PCR was conducted for the relative quantification of IL-1β, IL-8, IL-10, IL-23, and TGF-β. Briefly, 1 µg of total RNA was run for cDNA synthesis using random hexamers (Thermo Fisher Scientific, Inc. USA). Rotor-Gene Q (Qiagen, Hilden, Germany) was used for PCR amplification. Pre-denaturation at 95 °C for 10 min; 40 cycles of 15 s at 95 °C, 20 s at 58 °C, and 20 s at 72 °C were the amplification settings. GAPDH was used as the internal control^58^.

### Inflammatory signaling assay using western blotting and ELISA

The relative amount of phosphorylated NF-B protein was quantified by western blotting. After the treatments, macrophages were washed with ice-cold PBS. Total protein was extracted through a lysis buffer made up of 8 M urea and 2 M thiourea in 10 mM Tris at pH 8.0. Utilizing the Bradford assay, proteins were colorimetrically measured. On a 12% SDS-PAGE gel, 30 μg of the protein were electrophoresed, and a semi-dry electrotransfer (Bio-Rad, Richmond, CA) was used to blot the proteins on a nitrocellulose membrane. The membrane was blocked for 1 h with 5% BSA and then treated with the p-NF-κB antibody p65 overnight at 4 °C. Afterwards, over the next 2 h at room temperature, the secondary antibody (m-IgGκ BP-HRP with a 1:5000 v/v dilution) was applied. The protein-antibody combination was detected using a fluorescent blot scanner (Li-Cor, Lincoln, NE) and luminal solution. β-actin was the internal normalizer^59^. On the other hand, TNF-α and IL-6 were assayed using ELISA kits (Pars Gene, Iran) in the cell-free supernatants. 50 μl of the standards and macrophage supernatants were separately added to IL-6 or TNF-α coated wells. Plates were gently shacked at 200 rpm for 2 h at 37 °C. Then, 50 μl detection antibody was added to the wells. Plates were incubated at 37 °C for 1 h at 200 rpm. Following three rounds of washing, plates were incubated with avidin-HRP at 200 rpm for 30 min at ambient temperature. After thorough washing, the plates were incubated with the 3,3′,5,5′-tetramethylbenzidine (TMB) substrate solution for 15 min. Finally, 25 μl of stop solution was added, and the absorbance was recorded at 450 nm^60^.

### Statistical analyses

The findings were expressed as mean values ± standard deviation (SD) of at least three independent experiments and analyzed on GraphPad Prism 8. p-values < 0.05 were considered statistically significant.

### Ethical statement

The authors have read and have abided by the statement of ethical standards for manuscripts submitted to the journal. This article does not contain any studies with human participants or animals performed by any of the authors. We declare that the submitted manuscript does not contain previously published materials and are not under consideration for publication elsewhere. All the authors have made a substantial contribution to conception and design, or collection, analysis and interpretation of data, writing or revising the manuscript, or providing guidance on the research’s execution.

### Supplementary Information

Supplementary Information.

## Data Availability

The datasets generated and/or analyzed during the current study are available on request from the corresponding author.
