# Supplementary material for: Intelligent berberine-loaded erythrocytes attenuated inflammatory cytokine productions in macrophages
Source: Sci Rep. 2024 Apr 23;14:9381. doi: 10.1038/s41598-024-60103-9 (PMC11039762; doi:10.1038/s41598-024-60103-9)
Supplement: Supplementary file 1 — Supplementary Information. [file 41598_2024_60103_MOESM1_ESM.pdf]

Intelligent berberine-loaded erythrocytes attenuated inflammatory cytokine productions in macrophages

Zahra Sadat Aghili [1], Mauro Magnani [2], Mehdi Ghatrehsamani [3], Azar Nourian Dehkordi [3], Seyed Abbas Mirzaei [3,4*], Mehdi Banitalebi Dehkordi [3*]

[1] Department of Molecular Medicine, School of Advanced Technologies, Shahrekord University of Medical Sciences, Shahrekord, Iran

[2] Department of Biomolecular Sciences, University of Urbino Carlo Bo, Via Saffi 2, 61029 Urbino (PU), Italy

[3] Cellular and Molecular Research Center, Basic Health Sciences Institute, Shahrekord University of Medical Sciences, Shahrekord, Iran

[4] Department of Medical Biotechnology, School of Advanced Technologies, Shahrekord University of Medical Sciences, Shahrekord, Iran

Corresponding authors

Mehdi Banitalebi Dehkordi; Cellular and Molecular Research Center, Basic Health Sciences Institute, Shahrekord University of Medical Sciences, Shahrekord, Iran

Email: banitalebi.m@skums.ac.ir; mehdibanitalebi@gmail.com

Phone: (+98) 38 33335653; Fax: (+98) 38 33331471

Seyed Abbas Mirzaei; Cellular and Molecular Research Center, Basic Health Sciences Institute, Shahrekord University of Medical Sciences, Shahrekord, Iran. Email: mirzaei.a@skums.ac.ir; dr_amirzaei@yahoo.com. Phone: (+98) 38 33335653; Fax: (+98) 38 33331471

Supplementary Table S1. L16 Orthogonal array matrix in Taguchi experiment design

Taguchi Trial Number	Time	Temperature	pH	Drug concentration
1	1st Level: 15 Min	1st Level: 0 ºC	1st Level: 7	1st Level: 250 µg/ml
2	1st Level: 15 Min	2nd Level: 8 ºC	2nd Level: 7.4	2nd Level: 500 µg/ml
3	1st Level: 15 Min	3rd Level: 25 ºC	3rd Level: 7.8	3rd Level: 1000 µg/ml
4	1st Level: 15 Min	4th Level: 37 ºC	4th Level: 8.2	4th Level: 1500 µg/ml
5	2nd Level: 30 Min	1st Level: 0 ºC	2nd Level: 7.4	3rd Level: 1000 µg/ml
6	2nd Level: 30 Min	2nd Level: 8 ºC	1st Level: 7	4th Level: 1500 µg/ml
7	2nd Level: 30 Min	3rd Level: 25 ºC	4th Level: 8.2	1st Level: 250 µg/ml
8	2nd Level: 30 Min	4th Level: 37 ºC	3rd Level: 7.8	2nd Level: 500 µg/ml
9	3rd Level: 60 Min	1st Level: 0 ºC	3rd Level: 7.8	4th Level: 1500 µg/ml
10	3rd Level: 60 Min	2nd Level: 8 ºC	4th Level: 8.2	3rd Level: 1000 µg/ml
11	3rd Level: 60 Min	3rd Level: 25 ºC	1st Level: 7	2nd Level: 500 µg/ml
12	3rd Level: 60 Min	4th Level: 37 ºC	2nd Level: 7.4	1st Level: 250 µg/ml
13	4th Level: 120 Min	1st Level: 0 ºC	4th Level: 8.2	2nd Level: 500 µg/ml
14	4th Level: 120 Min	2nd Level: 8 ºC	3rd Level: 7.8	1st Level: 250 µg/ml
15	4th Level: 120 Min	3rd Level: 25 ºC	2nd Level: 7.4	4th Level: 1500 µg/ml
16	4th Level: 120 Min	4th Level: 37 ºC	1st Level: 7	3rd Level: 1000 µg/ml

(A) 100 µg/ml

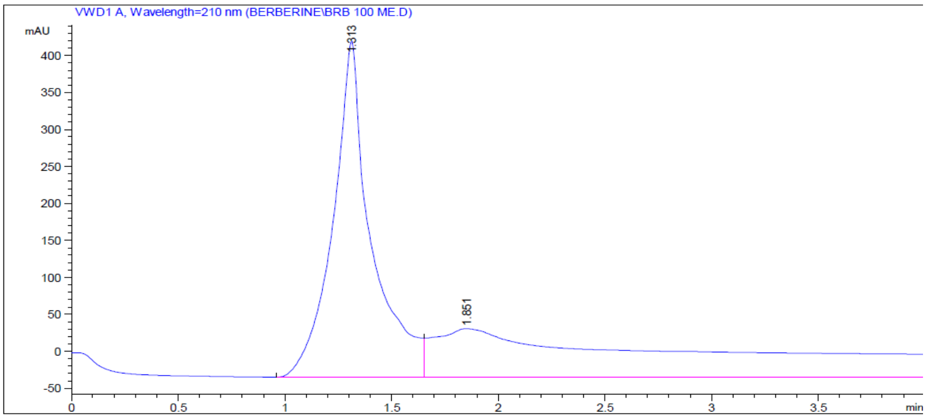

(B) 50 µg/ml

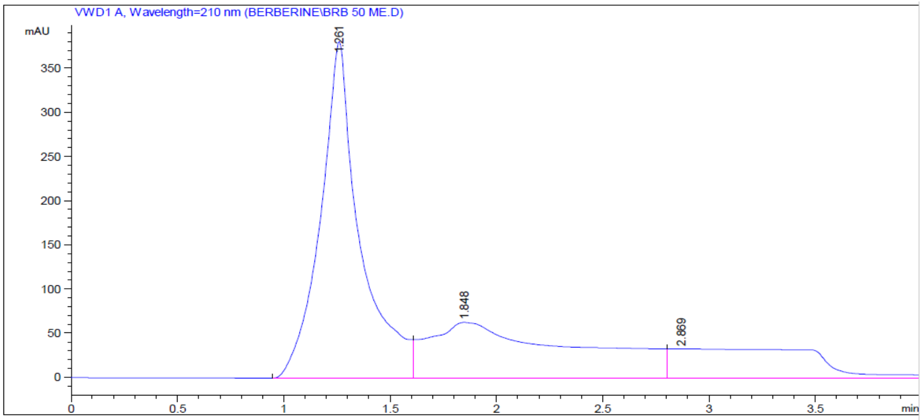

(C) 25 µg/ml

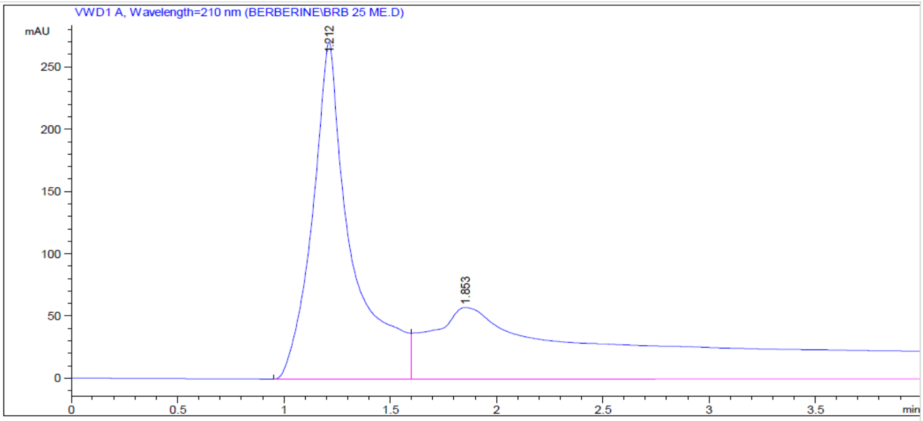

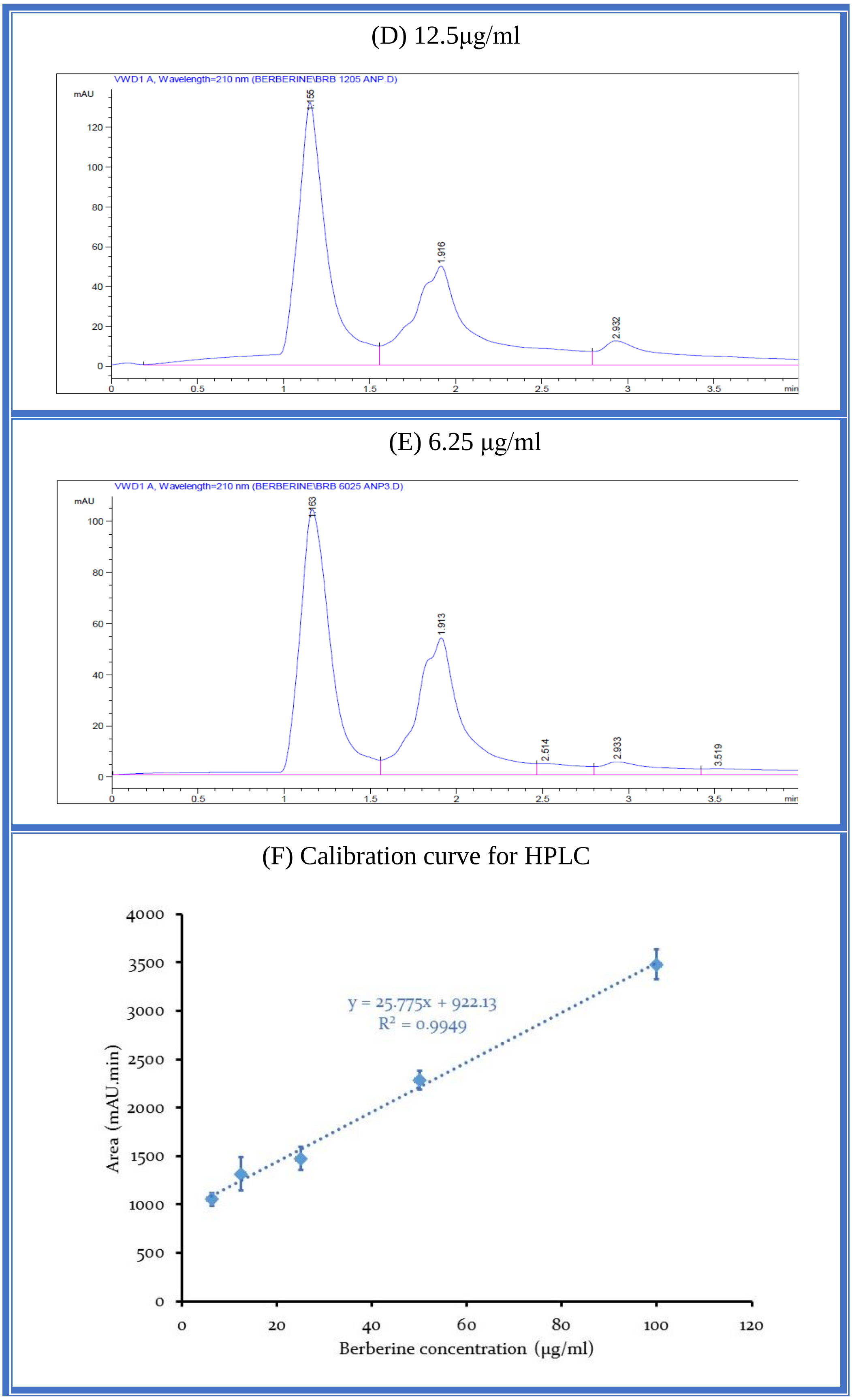

Supplementary Figure S1. *(*A-E) HPLC standard chromatogram showing retention peak of berberine around 1.2 min at wavelength of 210 nm over a range of 6-100 µg/ml.(F) Calibration

curve of HPLC showing the linearity of the data over a range of 6 to 100 µg/ml.Berberine was subjected to standard dilutions in acetonitrile, with the resulting graphs indicating an additional peak that corresponds to acetonitrile (≈1.9 min). Column chromatography was conducted on an Agilent HPLC (1100 series, Waldbronn, Germany) equipped with a UV detector (VWD, Agilent Technologies, set at 210 nm) and a C18 column (25 cm × 4.6mm, Agilent; injection volume 20 µl; flow rate of 1.0 ml/min).

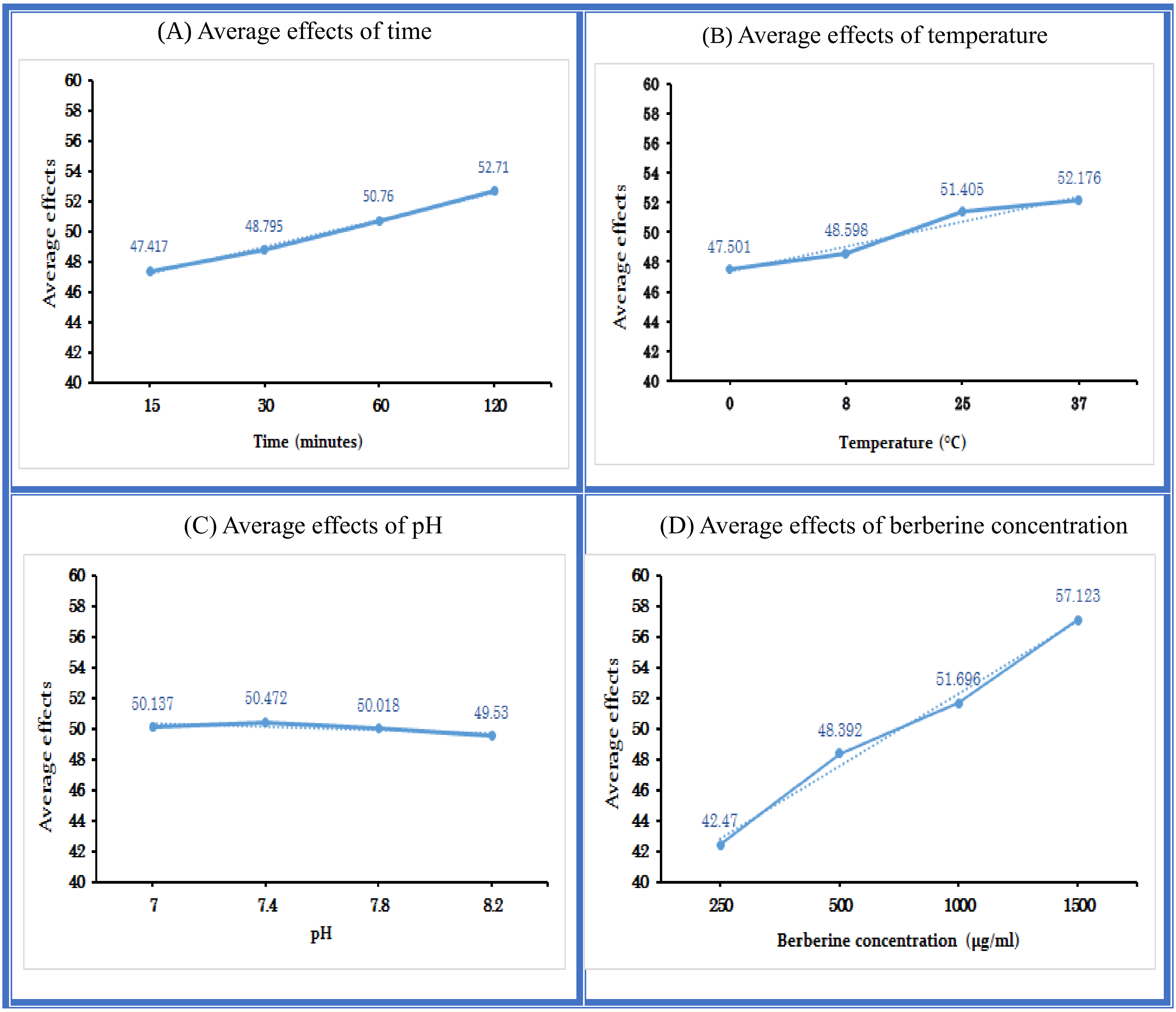

Supplementary Figure S2. Obtained plots from Qualitek-4 software that shows the effects of the selected factors on berberine loading in erythrocytes. The X-axis of the figure displays the various levels of the relevant factor, while the Y-axis displays the average influence of the relevant factors at the level. Berberine concentration showed highest substantial influence compared to other factors. Berberine loading improved with increases in berberine concentration, incubation temperature, and time. But pH levels has minimal effects on the loading responses.

Interaction between temperature and pH

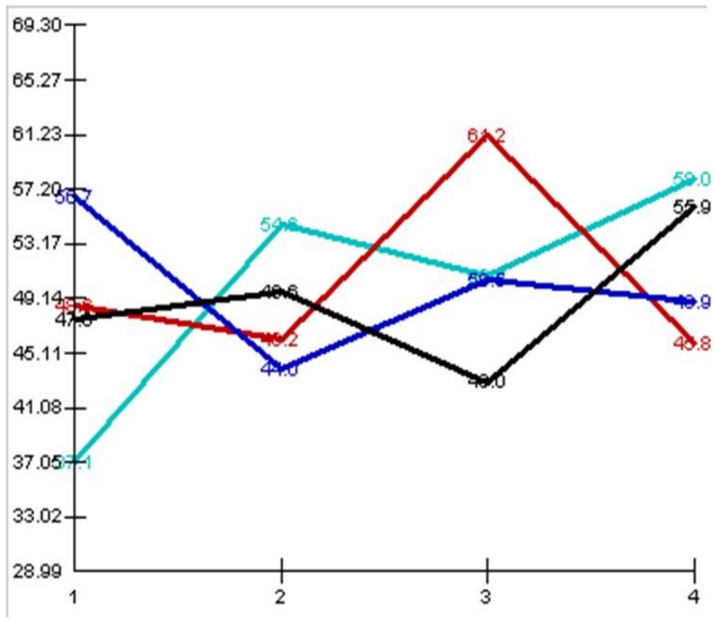

Levels of temperature

Severity Index: 41.41%
Optimum condition: [25°C, pH:7.4]
========================
X : Temperature
Y : pH

Plotted Values
==========================

X1Y1: 37.05 X3Y1: 50.87
X2Y1: 54.61 X4Y1: 58.01

X1Y2: 48.64 X3Y2: 61.23
X2Y2: 46.17 X4Y2: 45.84

X1Y3: 56.69 X3Y3: 50.49
X2Y3: 43.97 X4Y3: 48.9

X1Y4: 47.61 X3Y4: 43.01
X2Y4: 49.63 X4Y4: 55.94

Interaction between time and pH

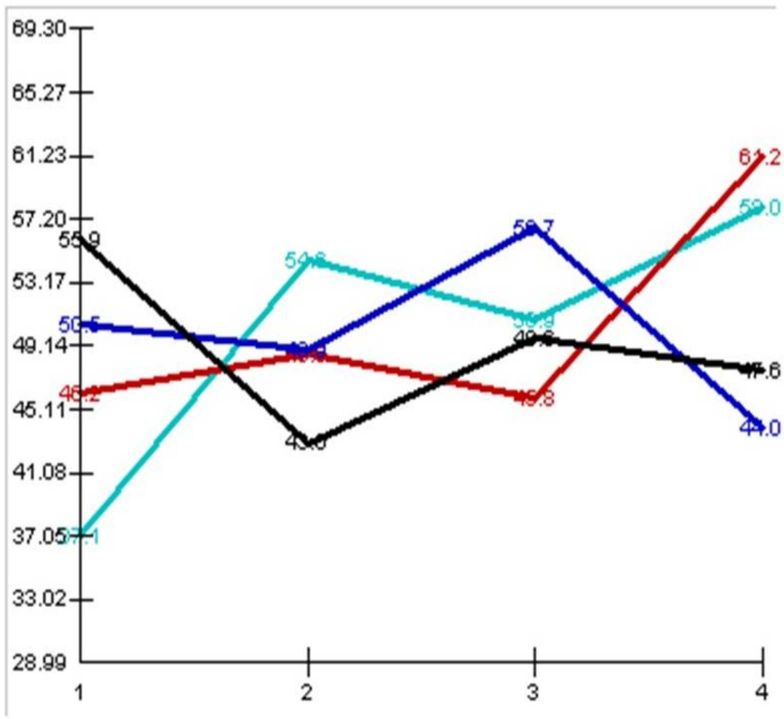

Levels of time

Severity Index: 31.21 %
Optimum condition : [120 min, pH:7.4]
========================
X : Time
Y : pH

Plotted Values
===========================

X1Y1: 37.05 X3Y1: 50.87
X2Y1: 54.61 X4Y1: 58.01

X1Y2: 46.17 X3Y2: 45.84
X2Y2: 48.64 X4Y2: 61.23

X1Y3: 50.49 X3Y3: 56.69
X2Y3: 48.9 X4Y3: 43.97

X1Y4: 55.94 X3Y4: 49.63
X2Y4: 43.01 X4Y4: 47.61

Interaction between pH and berberine concentration

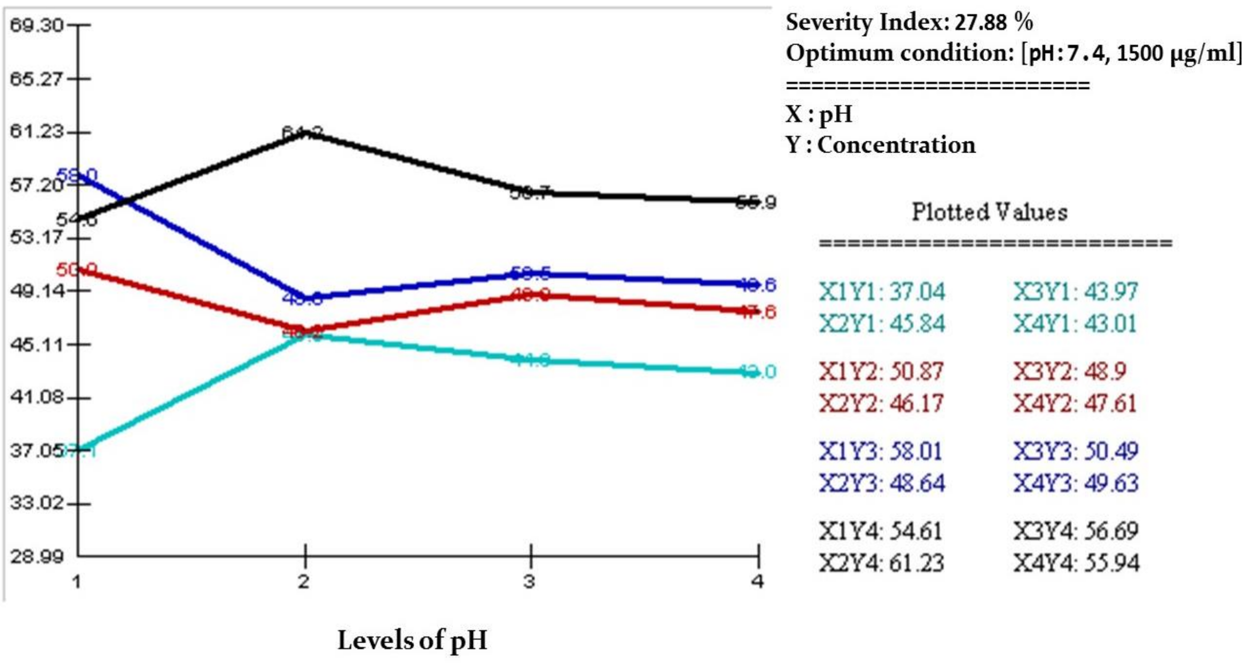

Interaction between temperature and berberine concentration

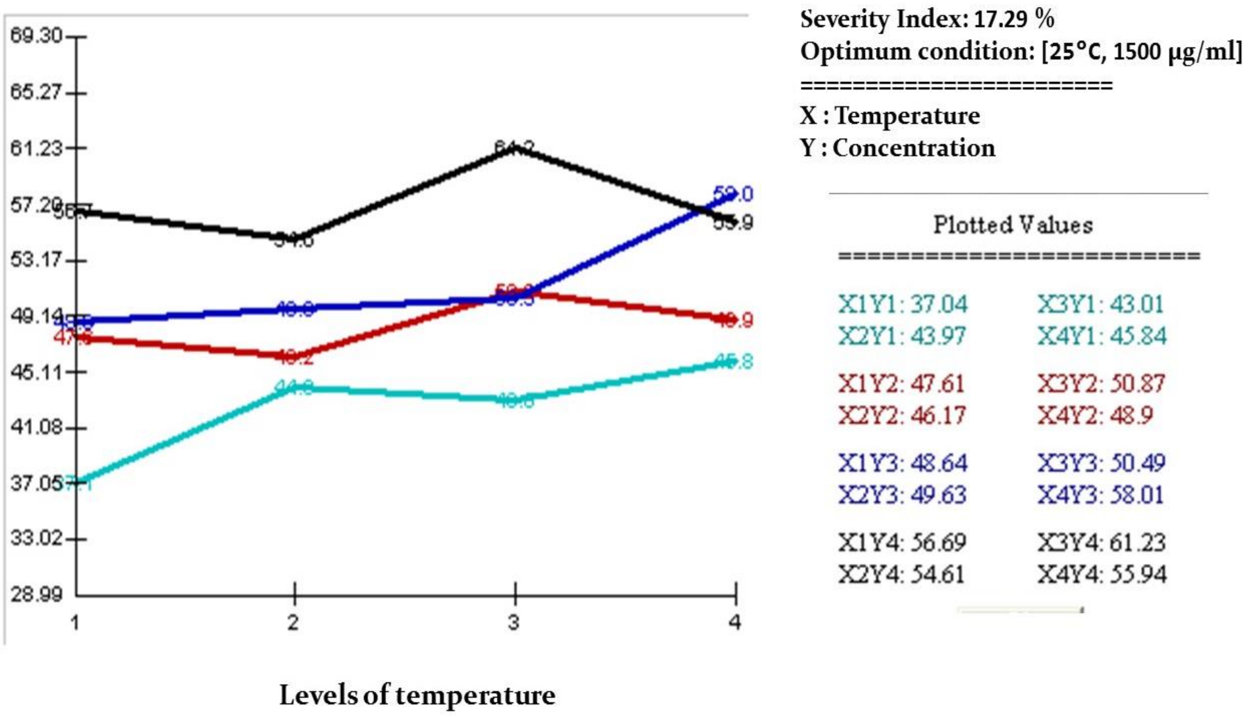

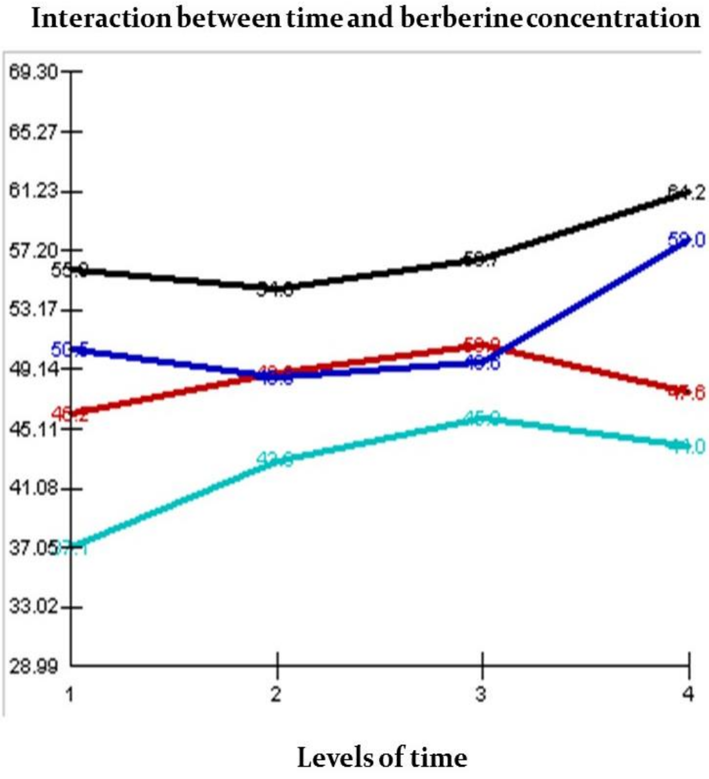

Severity Index: 6.68 %
Optimum condition: [120 min, 1500 µg/ml]
================================
X : Time
Y : Concentration

Plotted Values
==========================

X1Y1: 37.04	X3Y1: 45.84
X2Y1: 43.01	X4Y1: 43.97
X1Y2: 46.17	X3Y2: 50.87
X2Y2: 48.9	X4Y2: 47.61
X1Y3: 50.49	X3Y3: 49.63
X2Y3: 48.64	X4Y3: 58.01
X1Y4: 55.94	X3Y4: 56.69
X2Y4: 54.61	X4Y4: 61.23

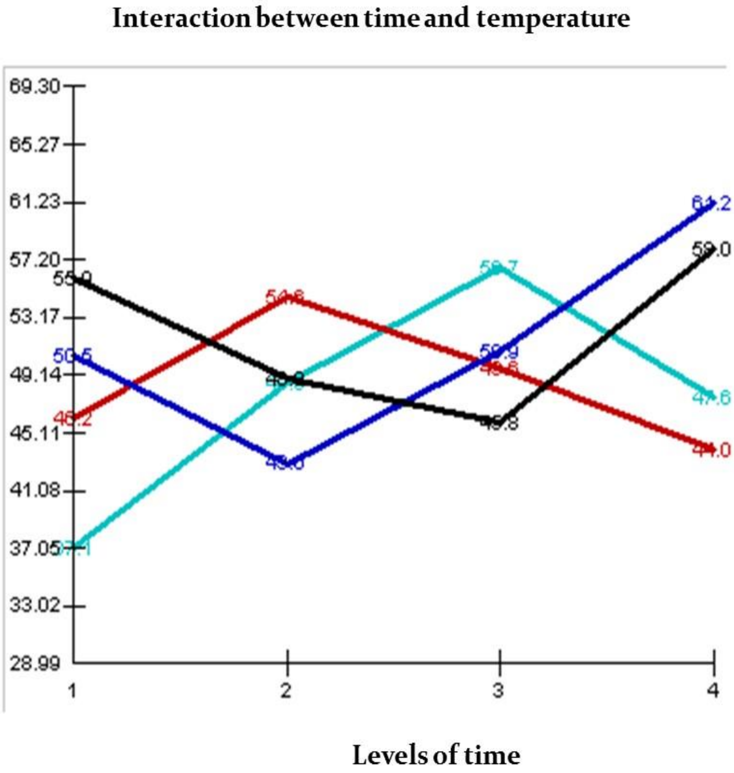

Severity Index: 6.5 %
Optimum condition: [120 min, 25°C]
===========================
X : Time
Y : Temperature

Plotted Values
===========================

X1Y1: 37.04	X3Y1: 56.69
X2Y1: 48.64	X4Y1: 47.61
X1Y2: 46.17	X3Y2: 49.63
X2Y2: 54.61	X4Y2: 43.97
X1Y3: 50.49	X3Y3: 50.87
X2Y3: 43.01	X4Y3: 61.23
X1Y4: 55.94	X3Y4: 45.84
X2Y4: 48.9	X4Y4: 58.01

Supplementary Figure S3. A plot obtained from Qualitek-4 software that shows main effect Interactions plot and severity index (SI) between selected parameters. The Interactions plot shows the mean effect of a selected factor versus another selected factor at each level and the highest predicted interactions of the given factors are depicted by a severity index.If a line breaks into 90 degrees then the severity index would be100%, in contrast parallel lines indicate a SI equal to 0%. The most significant elevated interaction, SI= 41.41%, was obtained between temperature and pH.Optimum condition: indicates the factor levels desirable for the optimum conditions (based strictly on the first two levels).

Supplementary Table S2. Analysis of Variance (ANOVA). Analysis of variance (ANOVA) was performed to determine the percentage (%) significance of every factor and to identify the factors that had a significant impact on the response value.

Factors	Degree of freedom (D)	Sum of squares(SS)	Variance(V)	F-Ratio(F)	Pure Sum(S´)	Percentage contribution (P)
Time	3	64.07	21.36	9.39	57.25	**9.76**
Temperature	3	59.57	19.86	8.73	52.75	**9.00**
pH	3	4.45	1.48	0.65	0	**0**
Drug concentration	3	451.51	150.51	66.21	444.70	**75.83**
Others/ Errors	3	6.81	--			**5.41**
Total	15	586.41	--			**100.00%**

The sum of the squares (SS), the degree of freedom (D), the variance (V), and the percentage of the contribution to the total variation (P) were used to formulate the ANOVA. Below is a description of the five parameter symbols that are frequently used in ANOVA:

1- The degree of freedom. D stands for the quantity of independent variables. If N is the total number of data points (or information) and M is the number of factor levels, then D_f (factor) corresponds to factor variance and D_e (error) relates to residual. The number of each parameter's levels, minus one, represents the parameter's degree of freedom:

$$D = D_f + D_e = M–1 + N–M = N–1$$

2- The sum of squares (SS) is a statistical measure used to calculate the variability or dispersion of a set of data points around their mean. SS_A represents the sum of squares of factor A, is calculated for a specific source of sum of squares as:

$$SS_A=\sum_{i=1}^{n}(x_i - \bar{x})^2$$

Where x is the mean or average of the data and xi is i-th answer or data.

3- Variance (V). When the total of squares is divided by the related degrees of freedom, the result is mean square or variance (V). Variance of a particular factor A, denoted as V_A, can be approximated by:

$V_A = \frac{SS_A}{D_A}$

4- The Fisher ratio (F) serves as the statistical measure employed to determine whether the means of the sample are situated within the scope of sampling variability and determines the meaningfulness of a factor. The presence of natural or random error variability can account for the occurrence of small values of S, whereas values of F exceeding one signify significant differences in mean or average values among certain groups. The mathematical expression is:

$F = \frac{V_A}{V_e}$

Where V_e is the variance error.

5. The contribution to the overall variance as a percentage (P). The symbol P_p stands for the proportion of the total variation of each particular parameter:

$$P(\%) = \frac{SS_A}{SS_T} \times 100$$

Where S_T is the total of the sum of squares and S'_A is pure sum of squares can be calculated from below equation:

$SS'_A = SS_A - D_A V_e$

Supplementary Table S3. Predicted optimal culture conditions and their contributions on the selected levels**.**

**Factors**	**Level Description**	**Level**	**Contribution**
Time (min)	120	4	2.79
Temperature (°C)	37	4	2.26
pH	7.4	2	0.55
Drug concentration (μg/ml)	1500	4	7.20
Total contribution from all factors			12.80
Current grande average of performance			49.92
Expected result at optimum condition			62.72

Transform the performance value from S/N to the original unitsby Qualitek Software based on the S/N ratio as below, here are the expected results:

$$\frac{S}{N} = -10\ Log\ (MSD) = 62.72$$

Or $MSD = 10^{\left[-\frac{(S/N)}{10}\right]} = 0.000001$

$$MSD = \frac{\left[\left(\frac{1}{y1}\right)^2 + \left(\frac{1}{y2}\right)^2 + \cdots + \left(\frac{1}{yn}\right)^2\right]}{n}$$

$$= \left[Avg.\left(\frac{1}{y1}\right)^2\right] = 1/Yexp^2$$

Or $Y(\text{expected}) = SQR\left(\frac{1}{MSD}\right)$

Expected performance in QC units

(Overall evaluation criteria) is:

$Y(\text{expected}) = 1367.73\ unit$

(Based on S/N= 62.72 at optimum)

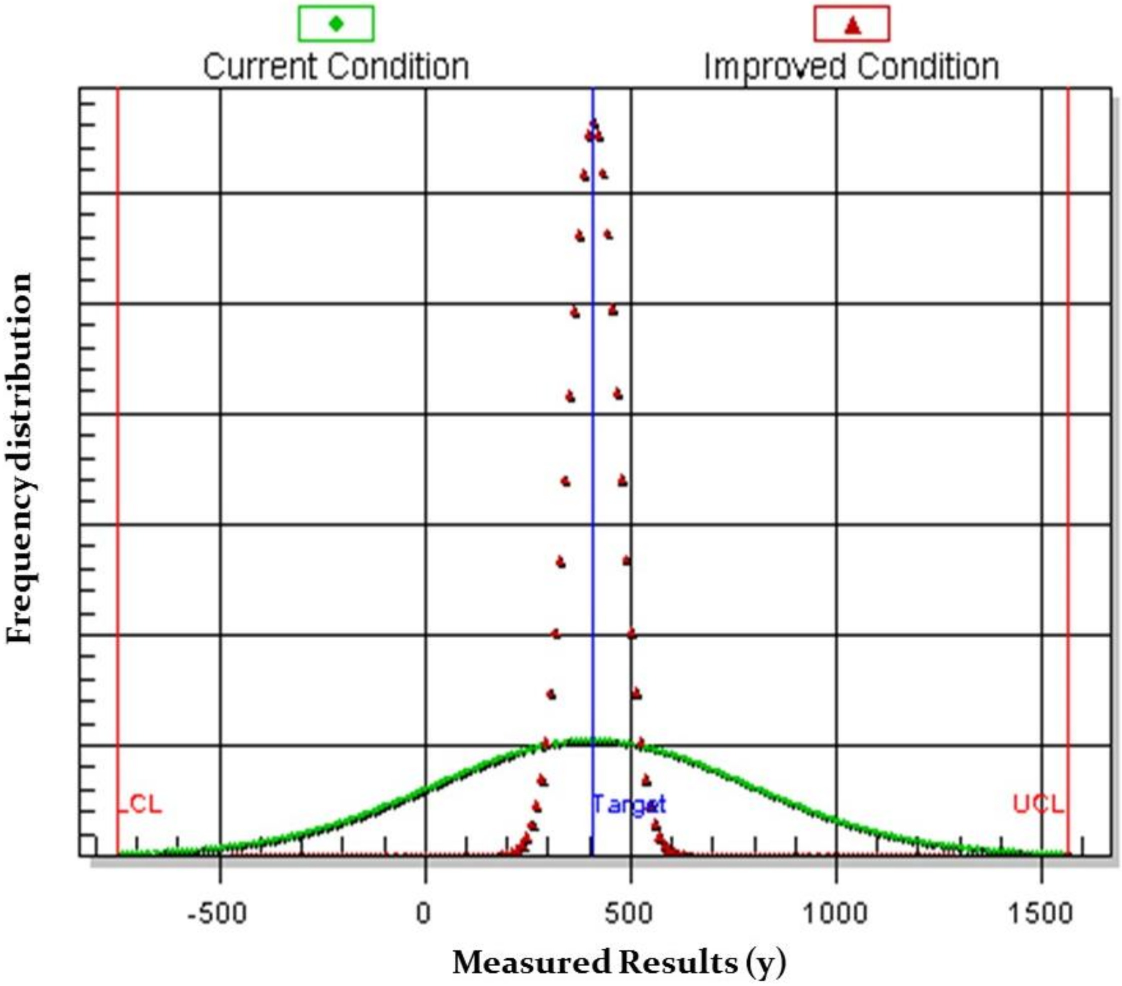

Plotting parameters	Current Condition	New/Improved Condition
**The Report S/N**	46.57	62.71
**average**	408.10	408.10
**Standard deviation**	384.88	59.96
**C_p**	1	6.42
**C_{pk}**	1	6.42
**Lower control limit (LCL)**	-746.55	-746.55
**Upper control limit (UCL)**	1562.75	1562.75

Supplementary Figure S4. Variation reduction plot shows performance distribution of berberine loading in erythrocytes in current and improved conditions. The variation reduction plot indicates the current and improved levels of drug loading based on the upper and lower

control limits (UCL and LCL). The steep peak on the graph represents reduced variation.The improved condition exhibits a smaller standard deviation in comparison to the current condition. The improved condition is based on minimizing the variance in the experimental response, whereas the present condition is obtained from the experimental response. As capability indices, Cp and Cpk values are used. Cp is a measure of process capability in relation to the difference between the UCL and LCL. Cpk is a statistical measure of variation in process relative to the mean. A high Cpk indicates that the process is capable of meeting its requirements. The capability index is higher in an improved condition instance than in the current situation. Taguchi's methodology involves quantifying quality through the discrepancy between a characteristic's target value and its actual value, and constructing a loss function [L(y)] to assess this discrepancy. The loss function is expressed as $L(y) = kx(y-m)^2$, where k represents the constant of proportionality, m denotes the target value, and y signifies the experimental value obtained after each trial.

(A) Ponceau-S staining on nitrocellulose membrane

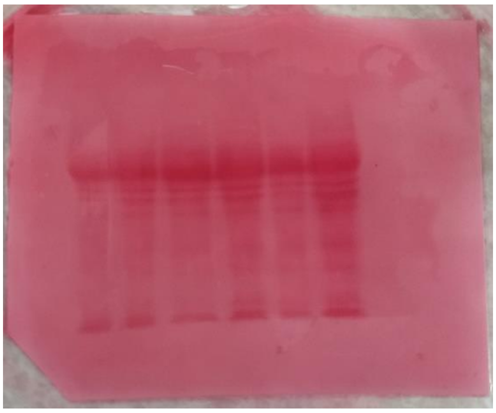

(B) Coomassie brilliant blue R250 staining after blotting

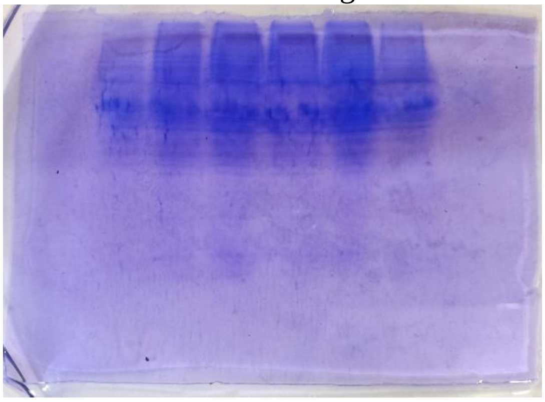

(C) Western analysis of β-actin (high exposure)

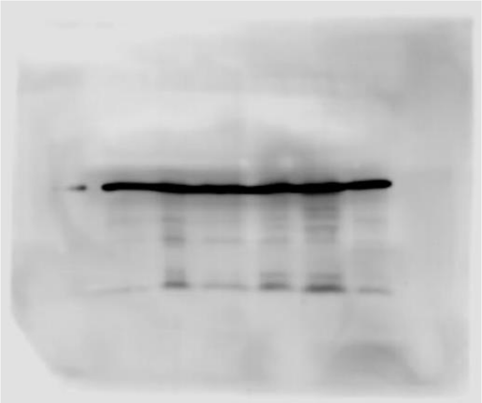

(D) Western analysis of β-actin (medium exposure)

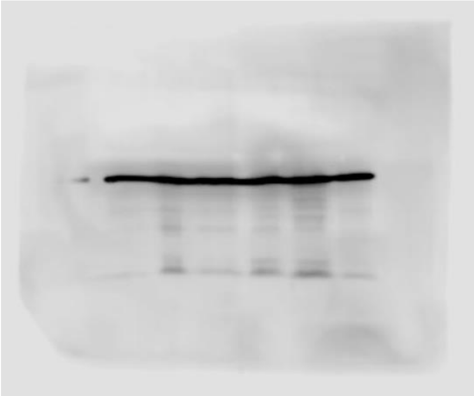

(E) Western analysis of β-actin (low exposure)

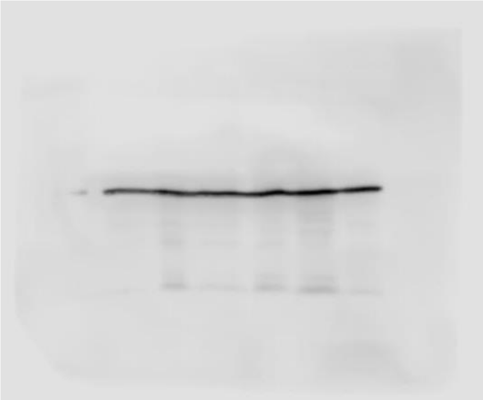

(F) Western analysis of NF-κB P65 (high exposure)

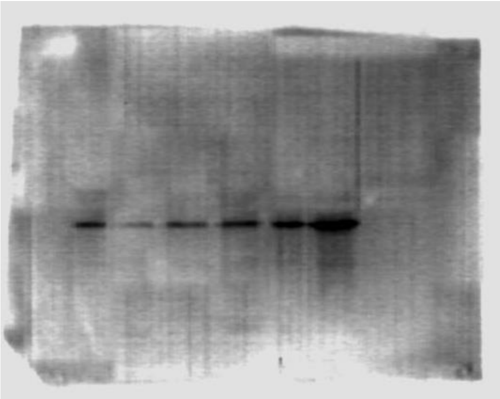

(G) Western analysis of NF-κB P65 (medium exposure)

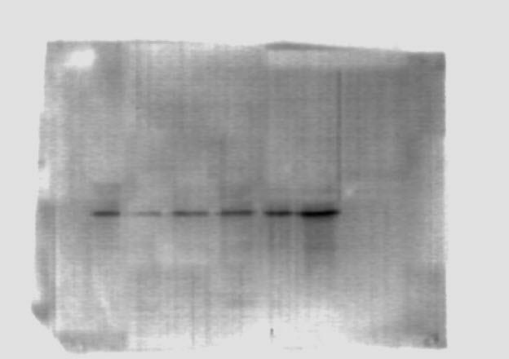

(H) Western analysis of NF-κB P65 (low exposure)

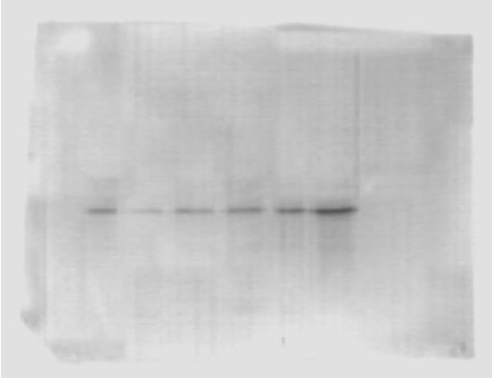

Supplementary Figure S5. Original pictures of ponceau-S staining (A), coomassie brilliant blue R250 staining of gel after transferring (B), and the western blots of β-actin from high exposure to low exposure (C-E), and NF-κB P65 from high exposure to low exposure (F-H). β-actin (Santa Cruz Biotechnology, sc-47778, a 45 kD protein band) was used as the normalizing protein. p-NF-κB P65 antibody (Santa Cruz Biotechnology, sc-136548, a 65 kD protein band) was the primary antibody and mouse IgG kappa binding protein conjugated to horseradish peroxidase (m-IgGκ BP-HRP, Santa Cruz Biotechnology, sc-516102) was the secondary antibody. Band positions from left to right are: undifferentiated U937 cells (lane 1), non-treated differentiated U937 cells as the control (lane 2), differentiated U937 cells treated with LPS and free berberine (lane 3), differentiated U937 cells treated with LPS and berberine-loaded erythrocytes (lane 4), differentiated U937 cells treated with LPS and non-loaded erythrocytes (lane 5), differentiated U937 cells treated with LPS (lane 6).

Supplementary Table S4. Primers used for real-time PCR analyses

Gene	Primer sequence	Lenght	Tm (ºC)
GAPDH	**Forward 5'-ACAGTCAGCCGCATCTTC-3'**<br>**Reverse 5'-CTCCGACCTTCACCTTCC-3'**	18<br>18	57.39<br>56.66
IL-1β	**Forward 5'-ACAGATGAAGTGCTCCTTCC-3'**<br>**Reverse 5'-GTCGGAGATTCGTAGCTGGAT-3'**	20<br>21	57.22<br>59.36
IL-8	**Forward 5'-TTGGCAGCCTTCCTGATTTC-3'**<br>**Reverse 5'-TATGCACTGACATCTAAGTTCTTTAGCA-3'**	20<br>28	58.45<br>60.79
IL-10	**Forward 5'-TCAAGGCGCATGTGAACTC-3'**<br>**Reverse 5'-CGGCCTTGCTCTTGTTTTC-3'**	19<br>19	58.46<br>57.52
IL-23	**Forward 5'-TCAGTGCCAGCAGCTTTCAC-3'**<br>**Reverse 5'-TCTCTTAGATCCATGTGTCCCAC-3**	20<br>23	61.17<br>59.29
TGF-β	**Forward 5'-CAGCAACAATTCCTGGCGATA-3'**<br>**Reverse 5'-AAGGCGAAAGCCCTCAATTT-3'**	21<br>20	58.98<br>58.37
